# An upgraded nuclease prime editor platform enables high-efficiency singled or multiplexed knock-in/knockout of genes in mouse and sheep zygotes

**DOI:** 10.1093/procel/pwaf006

**Published:** 2025-01-20

**Authors:** Weijia Mao, Pei Wang, Lei Zhou, Dongxu Li, Xiangyang Li, Xin Lou, Xingxu Huang, Feng Wang, Yanli Zhang, Jianghuai Liu, Yongjie Wan

**Affiliations:** Livestock Embryo Engineering Laboratory, College of Animal Science and Technology, Nanjing Agricultural University, Nanjing 210095, China; State Key Laboratory of Pharmaceutical Biotechnology and MOE Key Laboratory of Model Animals for Disease Study, Model Animal Research Center at Medical School of Nanjing University, Nanjing 210061, China; Livestock Embryo Engineering Laboratory, College of Animal Science and Technology, Nanjing Agricultural University, Nanjing 210095, China; Livestock Embryo Engineering Laboratory, College of Animal Science and Technology, Nanjing Agricultural University, Nanjing 210095, China; The Key Laboratory of Pancreatic Diseases of Zhejiang Province, The First Affiliated Hospital, Zhejiang University School of Medicine, Hangzhou 310003, China; Zhejiang Laboratory, Hangzhou 311121, China; The Key Laboratory of Pancreatic Diseases of Zhejiang Province, The First Affiliated Hospital, Zhejiang University School of Medicine, Hangzhou 310003, China; School of Life Science and Technology, ShanghaiTech University, Shanghai 201210, China; Livestock Embryo Engineering Laboratory, College of Animal Science and Technology, Nanjing Agricultural University, Nanjing 210095, China; Livestock Embryo Engineering Laboratory, College of Animal Science and Technology, Nanjing Agricultural University, Nanjing 210095, China; State Key Laboratory of Pharmaceutical Biotechnology and MOE Key Laboratory of Model Animals for Disease Study, Model Animal Research Center at Medical School of Nanjing University, Nanjing 210061, China; Livestock Embryo Engineering Laboratory, College of Animal Science and Technology, Nanjing Agricultural University, Nanjing 210095, China

**Dear Editor,**

The emergence of CRISPR/Cas9-based genome editing technology has energized many applied biotechnology fields, including livestock breeding (Zhao et al., 2019). Given that the most naturally occurring beneficial variants in farm animals are regulatory alleles (Georges et al., 2019), practical livestock editing should mostly involve knocking-in of the natural variants, or of designed elements to regulate key genes. However, earlier CRISPR/Cas9-dependent strategies for installation of knock-in alleles showed limitations of low efficiencies and purities (Anzalone et al., 2020).

An important breakthrough toward precise genome editing was marked by the development of prime editor (PE), which presented a highly innovative design for the installation of small-sized edits (Anzalone et al., 2019). The canonical PE protein features a fusion of Cas9 nickase (nCas9, H840A) moiety and a reverse transcriptase (RTase) domain. A prime editing guide RNA (pegRNA) is comprised of a conventional sgRNA module (for initially directing a nick at the target site) and an additional 3′ sequence extension (for subsequently programming synthesis of edits at the nick by reverse transcription (RT)). The resultant intermediate 3′-flap structure may be subsequently resolved by the cellular repair pathways for the eventual installation of edits (Anzalone et al., 2019). Empowered by such a double-strand DNA break (DSB)-independent and RT-dependent mode of action, PE has exhibited high editing versatility and genomic safety in diverse application contexts (Chen and Liu, 2023). On the other hand, PE’s editing efficiencies have often been sub-optimal and inconsistent, presenting a technical challenge to inspire continuous enhancement efforts (Chen and Liu, 2023). An important sub-class of such efforts centered on the development of nuclease PE (PEn, based on the nuclease-active Cas9, Fig. S1) that could increase the rates of desirable edits at some sites (Li et al., 2023). To upgrade PEn toward more efficient installation of precise edits, we recently adopted an accessory protein regulator of DSB repair [a ubiquitin variant, also called i53 (Canny et al., 2018)] to establish a high-activity uPEn platform (Li et al., 2023). The potent activities of uPEn presented exciting opportunities, despite that it remained liable for a fraction of DSB-derived editing impurities (Li et al., 2023). We envisioned that the cutting-edge PE/PEn tools could be suited for the installation of defined, beneficial genome edits in livestock. *PPARG* (encoding PPARγ, a lipogenic transcription factor) represents an attractive target for gain-of-function editing, due to its association with intramuscular fat-dependent meat quality traits (Liu et al., 2020). For proof-of-principle, the present study would aim to precisely edit *PPARG* in Hu sheep, a major Chinese indigenous sheep breed with a high reproduction rate and commendable meat production (Yue, 1996).

We first adapted the canonical PE protein component into a very recent “PEmax” architecture (with several elements of optimization over the initial PE (Chen and Liu, 2023)). Furthermore, several split forms of the PEmax were constructed similarly as in a previous study (Liu et al., 2022) (Fig. S2A). The basic split design featured separate nCas9 and RTase parts. In another variant split form, the non-essential RNase-H domain was further removed from the RTase [ΔRH] (Chen and Liu, 2023). In parallel, a GCN4 tag (1×) and the corresponding single-chain fragment variable (scFv) binder were respectively placed on the nCas9 and RTase (with or *w*/*o* the RH) moieties to potentiate their interactions (Liu et al., 2022). The activities of various PEmax forms were tested in human HEK293T cells for disabling a premature TAG stop codon within a reporter construct (Fig. S2A). Note that all pegRNA constructs in the present study were assembled with an enhancement design of 3′ xrRNA-joining, in accordance with our previous work (Zhang et al., 2022). The results of reporter editing showed that the full-length PEmax and the split/tagged form with the RTaseΔRH (PEmax-SPLΔRH-tag) were overall the top performers, followed by the SPL-tag and SPLΔRH forms with slightly lower activities (Fig. S2B and S2C), while the modest differences in activity patterns of different PE forms were apparently not attributed to their expression levels (determined by immunoblotting of nCas9, Fig. S2D). We also determined the efficiencies of different PEmax formats for editing three previously tested genomic loci in HEK293T cells (Zhang et al., 2022). Herein, the locus-dependent activities of the four different split PE forms were comparable with each other, while they were modestly lower than those of the full-length form (Fig. S2E). These results suggested the possibility of exploiting the editors in either the full-length or split format for later testing.

We next considered to design the modification at *PPARG*. The *PPARG* locus encodes two overlapping PPARγ isoforms with distinct promoters and translational start sites (i.e., broadly expressed γ1 and adipose-specific γ2 isoforms) (Hu et al., 2022) (Fig. S3A). Therefore, editing at γ2-specific portion of *PPARG* would selectively impact the adipocytes, and conceivably avoid a global effect. Interestingly, a closer examination of the sequences revealed that the Kozak motifs preceding mouse *Pparg* (γ2) and sheep *PPARG* (γ2) start codons deviate substantially from the consensus sequence of “GCCACC” (Kozak, 1987) (Fig. 1A). Thus, a knock-in allele with a favorable Kozak motif preceding the start codon of *PPARG* (γ2, mouse, and sheep) could potentially specify higher PPARγ2 expression. To this end, an initial trial was designed for precise Kozak motif replacement in mouse cells. Note that the suitable break position is located relatively distant (30 bp) from the Kozak sequence at the mouse target (an issue similarly present at the sheep target) (Fig. S3B), which is likely to undermine PE efficiencies (Anzalone et al., 2019). Indeed, although the guide RNA target site was susceptible to cleavage by Cas9/sgRNA (Fig. S4A), we found that PEmax in combination with the pegRNA for replacement editing (“pegRNA-M-replc”, Table S1) failed to induce desirable sequence changes in mouse N2a cells (Fig. S4B and S4C).

**Figure 1.**
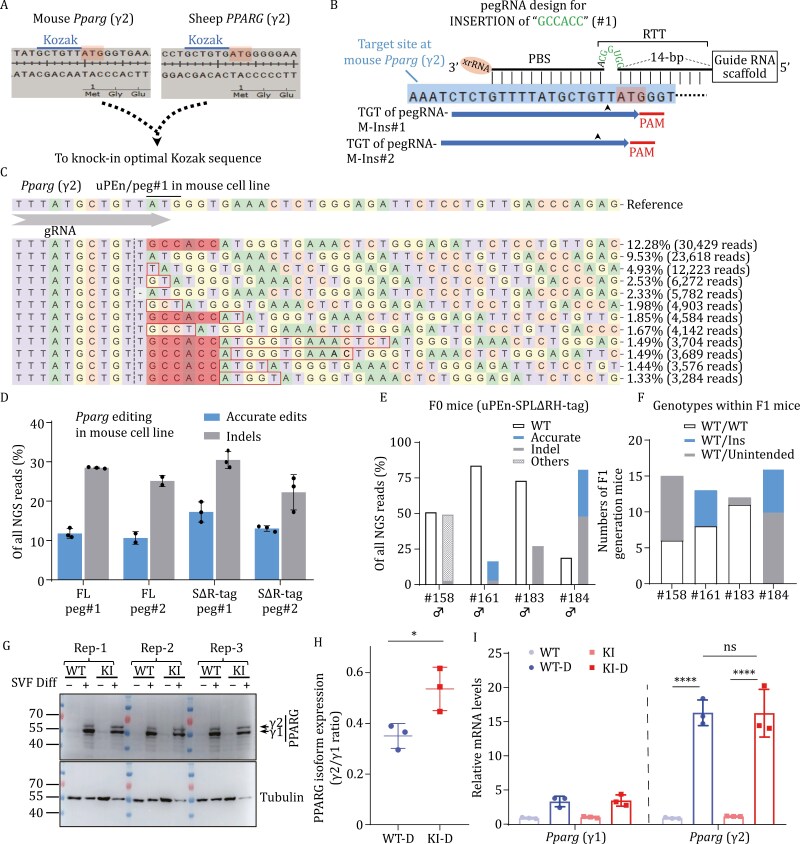
**uPEn-dependent knock-in of an optimal Kozak sequence in mice.** (A) Sequences near the start codon (the "ATG" in the middle portion of the top strand) of mouse and sheep *PPARG* (γ2). The original Kozak motifs (marked) apparently deviate from the consensus. (B) Designs of pegRNAs to insert a consensus Kozak motif upstream of mouse *Pparg* (γ2) are illustrated. Two overlapping target sites can be selected, corresponding to pegRNA-M-Ins#1 and pegRNA-M-Ins#2. The respective PAMs ("GGG" and "TGG"), the guide RNA target sequences (rightward thick arrows), and the cleavage points (upward arrowheads) are marked. The xrRNA protective module, PBS, and RTT for pegRNA-M-Ins#1 are also schematically shown on top of the target sequence. The "CGGUGG" RNA sequence corresponds to the consensus Kozak motif to be inserted directly upstream of the start codon. (C and D) The mouse N2a cell line was transfected with different uPEn formats and pegRNAs. The DNA samples from the edited cells were subjected to NGS analyses. In (C), corresponding to an editing sample from cells transfected with FL uPEn and pegRNA-M-Ins#1, the allele distribution graph is shown with the top reads. The shaded sequences highlight Kozak motif insertion. In (D), the quantifications of accurate modifications and indels in different editor groups are presented. (E) Quantifications of next-generation sequencing results (mouse *Pparg* locus) on toe clip samples from individual F0 founder mice are presented. For each sample, the percentage of the WT allele is shown by the left column, while those of edited alleles are presented by the right column. Different types of edits are indicated with different shades or patterns. (F) The founder mice were bred with WT mice to obtain the F1 generation. The toe clips were subjected to Sanger sequencing. The genotype distributions within all F1 progenies of the founder mice (#158, #161, #183, and #184) are shown in the graph. Overall, the distribution of F1 genotypes correlated with the allele types and frequencies of the founder mice. (G–I) The SVF cells isolated from the subcutaneous inguinal adipose tissue of the WT and the homozygous knock-in (KI) mice were subjected to *in vitro* adipogenic induction. Each genotype/condition was represented by three replicate wells. On day-7 following induction, the protein/RNA samples were harvested. The Western blot results are presented (G). The ratios of PPARγ2/PPARγ1 levels (± SD) were also determined. Quantitation was based on averaged values from three independent experiments (each with two to three biological replicates) (H). Two-sided Student’s *t*-tests were used to determine the statistical differences between genotypes (*: *P* < 0.05). The differentiated cells from the WT and KI mice are denoted as “WT-D” and “KI-D,” respectively. (I) The RNA samples from triplicates of differentiated adipocytes were subjected to qPCR analyses (see Table S6 for primer information). The levels of *Pparg* (γ1 and γ2) mRNA were normalized to those of 36B4 (± SD). Statistical significance was determined using two-way ANOVA (****: *P* < 0.0001).

Alternatively, based on the positions of available “NGG” protospacer adjacent motifs (PAMs), we next considered insertion (not replacement) of an optimal Kozak directly upstream of ATG, which could be programmed via small sequence insertion after the + 1 or + 2 position in reference to the ^↓^TATGG or ^↓^TTATGG DNA break positions (Fig. 1B). A similar design could also be applied for the sheep site (see later section, Table S1). Moreover, with a mouse *Pparg* (γ2) upstream sequence-dependent reporter assay, we validated that the GCCACC-inserted construct drove higher reporter expression (Fig. S5A–C). Besides rational allele design to accommodate prime editing, we also chose to exploit our recently developed high-activity uPEn platform (Li et al., 2023). According to our earlier reporter- and genomic loci-editing characterizations on the formats of PE (Fig. S2), either the full-length uPEn or its split/tagged form with the RTaseΔRH (uPEn-SPLΔRH-tag) was applied in further experiments (Fig. S6A). Different combinations of two uPEn formats and two pegRNAs (respectively, corresponding to cleavages at ^↓^TATGG and ^↓^TTATGG [pegRNA-M-Ins#1 and #2], see Fig. 1B) were co-transfected into mouse N2a cells. The Sanger sequencing and next-generation sequencing (NGS, primes listed in Table S2) results clearly indicated the occurrence of Kozak motif insertion in all experimental groups (10%–17% in all NGS reads), despite the concomitant presence of editing impurities (Figs. 1C, 1D and S6B). With both the full-length and SPLΔRH-tag uPEn, pegRNA-M-Ins#1 showed a trend of being relatively more active between the two pegRNAs for introducing accurate edits and indels (Fig. 1D).

Given the positive results in cultured cells, we next proceeded to test the knock-in of the consensus Kozak motif at *Pparg* (γ2) in mouse zygotes. The uPEn-SPLΔRH-tag format was exploited. The mRNAs for Cas9-GCN4 and scFv-RTaseΔRH, and the pegRNA component (pegRNA-M-Ins#1) were, respectively, prepared by *in vitro* transcription (IVT, see Table S3 for primer information). The C57/BL6JGpt 1-cell stage embryos (100) were microinjected with the editor mix (97.5 ng/μL of Cas9-GCN4 and 52.5 ng/μL of scFv-RTaseΔRH mRNA, and 50 ng/μL of pegRNA) in the cytoplasm, and were subsequently transferred to foster mothers. Out of 49 mice born, four showed clear modifications at the target site, upon initial genotyping of the toe clips (Fig. S7A). Targeted NGS of samples from the clearly modified mice assigned two F0 mice (#161 and #184) as founders with accurate knock-in modifications (desirable allele representing ~14% and 33% of total reads, respectively) (Figs. 1E and S7B). The #184 mouse was shown to also bear a high rate of indel allele (46%), and only a low percentage of WT allele (18.9%). The other two F0 mice presented either an imprecisely inserted allele (#158, ~46%) or a non-specific indel (#183, ~25%) (Figs. 1E and S7B). The edited F0 mice were subsequently bred with the WT mice to produce the F1 generation cohorts, in which all mutant allele types demonstrated evident germline transmissions (Figs. 1F and S7C). To understand the apparent lack of transmission of the WT allele by the #184 mouse (in > 15 progenies), we harvested the tissues from testis and several other internal organs from the aged F0 for subsequent NGS analyses. The result suggested that cells bearing a WT allele were comparatively under-represented in the germ cell compartment (Fig. S7D), indicated by a group-low, 4% allele frequency in the testis sample (8.4%–27% in other tissues).

Further crossing of the heterozygous F1 mice (derived from the #161 founder) led to the delivery of homozygous knock-in progenies (Fig. S7E). A homogeneous population of adipocytes was prepared via differentiation of the stromal vascular fraction cells from the mice (Fu et al., 2023). Comparisons between the control and differentiated cells regardless of the genotypes confirmed that PPARγ (both γ1 and γ2 isoforms) protein levels were markedly upregulated under the adipogenic condition (Fig. 1G). Importantly, the cells (differentiated) with Kozak knock-in at *Pparg* (γ2) showed higher normalized protein expression for PPARγ2, but not PPARγ1, than their WT counterparts (Fig. S7F). This apparently led to a greater PPARγ2/γ1 ratio in the knock-in cells (Fig. 1H). In contrast, there were no genotype-dependent differences at the levels of *Pparg* γ1/γ2 mRNAs (Fig. 1I). Although an immediate survey of common PPARγ target genes did not show evident differences between the WT and knock-in cells (Fig. S7G), future examinations on potential changes driven selectively by PPARγ2 are warranted (Hu et al., 2022).

The above success in the mouse trial encouraged us to further apply uPEn to editing the Hu sheep zygotes. Programmed multi-locus edits in sheep would be a desirable strategy for balanced improvements of both meat production and meat quality traits (Zhao et al., 2019). Accordingly, we considered applying a mix of sgRNA/pegRNA to simultaneously program knockout at *MSTN* (Zhao et al., 2019) and knock-in at *PPARG* (Liu et al., 2020) (Fig. 2A). To this end, a pegRNA was designed for Kozak motif insertion directly upstream of ATG at sheep *PPARG* [γ2] locus (Fig. S8A), similar to the mouse site-targeting strategy (Fig. 1B). To ensure efficient knockout of *MSTN*, two sgRNAs targeting the same essential exon (exon 2) were designed (Fig. S8B). The trial was also set to use different formats of uPEn (full-length and SPLΔRH-tag forms).

**Figure 2.**
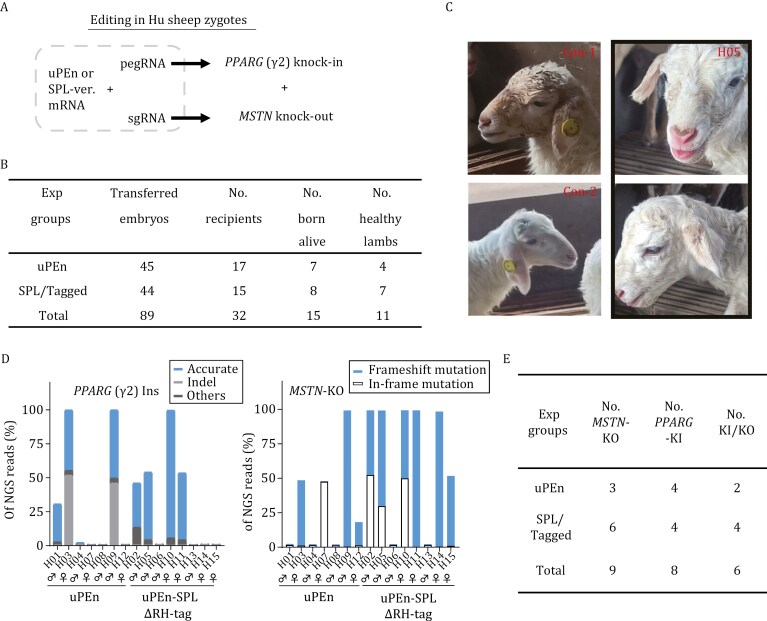
**Efficient two-locus knock-in/knockout modifications by uPEn in the Hu sheep.** (A) The strategy for dual-gene editing of Hu sheep is illustrated. (B) Following zygote injection, the cleaved embryos were transplanted to the surrogate ewes. In ~150 days, after the lambs were born, ear punches from the newborns were harvested. The numbers of the embryos, the surrogate ewes, and the total newborns from this experiment (two editing groups) are shown. (C) The images of two normal lambs and an experimental lamb are shown. The experimental lamb (H05) presented a phenotype of an enlarged tongue, suggesting its *MSTN* deficiency. (D) Quantitation of NGS results (left: *PPARG* locus, and right: *MSTN* locus) on ear punch samples from individual newborn lambs. For each sample, the percentage of different types of edited alleles is presented in different colors. (E) The overall numbers of newborn lambs with desirable knock-in and/or knockout alleles are presented.

The pegRNA, sgRNAs, and the mRNAs for two uPEn formats were prepared by IVT. The fertilized eggs of Hu sheep were microinjected (in cytoplasm) with the RNA mix (100 ng/μL of uPEn (or 65 ng/μL of Cas9-GCN4 and 35 ng/μL of scFv-RTaseΔRH) mRNA, 50 ng/μL of pegRNA, and 25 ng/μL of mixed sgRNAs), followed by embryo transfer. Five months later, a total of 15 F0 lambs were born (Fig. 2B). Interestingly, one of the F0 lambs presented obvious macroglossia (enlarged tongue) (Fig. 2C), a phenotype likely indicative of muscle hyperplasia related to *MSTN* editing. DNA samples from all newborns were subjected to targeted NGS at the *PPARG* and *MSTN* sites (Fig. S9A and S9B). For the uPEn group (*n* = 7) and the uPEn-SPLΔRH-tag group (*n* = 8), a major portion (4 and 4) of the newborns from each group presented accurate *PPARG* knock-in alleles (allele frequencies > 1%) (Figs. 2D, 2E and S9A). In the corresponding positive newborns of the two groups (H1, H3, H4, H9 and H2, H5, H10, H11, respectively), the average frequencies of the knock-in alleles were 33% and 62%, respectively. Among these knock-in-positive founders, one (H2) also presented an evident level (~10%) of imperfect prime edit, and two others (H3 and H9) harbored undesirable indels at the *PPARG* site (Figs. 2D and S9A), the latter apparently attributed to the Cas9 nuclease module in uPEn. Moreover, at the *MSTN* locus, 3 newborns from the uPEn group and 6 from the uPEn-SPLΔRH-tag group were shown to bear knockout edits (1 more in the uPEn group featuring in-frame deletions) (Figs. 2D, 2E and S9B). In total, an impressive number of F0 newborns (together 6 out of 15) presented desirable two-locus knock-in/knockout modifications (2 from uPEn group: 1 unfortunately deceased after birth, and 4 from uPEn-SPLΔRH-tag group) (Fig. 2E). Although the present proof-of-principle experiment is not sufficient for quantitative comparisons between the full-length and the SPLΔRH-tag uPEn formats on zygote editing, our results have evidently demonstrated two active forms of uPEn that might accommodate different application needs. Besides the on-target editing assessments, several potential genomic off-target sites for the pegRNA (*PPARG* editing, see Tables S4 and S5 for sequence and primer information) and the sgRNA (corresponding to the more active sgRNA2 against *MSTN*, see Fig. S8) were amplified using the samples from the edited founders, and those from three lambs presenting no on-target modifications (H7, H8, and H13). The comparisons of the NGS reads from edited and non-edited animals showed minimal off-target editing at the sites tested (Fig. S10), although further comprehensive analyses would be encouraged. In the meantime, we envision that future employment of high-fidelity Cas9 variants (reviewed in (Anzalone et al., 2020)) in uPEn may formally improve its genetic safety.

Collectively, extending on our recent development of an upgraded PE nuclease (uPEn) (Li et al., 2023), the present study has further demonstrated its effectiveness in installing small knock-in edits in mice, and on enabling multiplexed knock-in/knockout edits in Hu sheep zygotes. The validated improvement of PPARγ2 expression in cells from the Kozak element-edited mice has suggested our editing strategy as a widely applicable approach to program target gene expression in animals. Moreover, the observed high rate of simultaneous *PPARG*/*MSTN* knock-in/knockout edits in Hu sheep (despite certain evidence of stochastic, single-site editing) has highlighted uPEn’s potential for multi-target applications toward rapid and balanced livestock genetic improvements (Georges et al., 2019). In our two-site-targeting experiment, the *MSTN*-editing efficiencies by uPEn/sgRNA [10/15] duly served as a high-end benchmark to underscore the strong performances of uPEn/pegRNA-dependent Kozak insertion at *PPARG* [8/15 achieved] despite the expected activity restraints on pegRNA-driven editing owing to mechanistic complexities. One other technical aspect for future application of uPEn is whether chemically synthesized/modified pegRNA or IVT pegRNA (with 3′ stabilizing RNA structure) would be the preferred format. As an effort for direct comparison, we requested commercial synthesis (with HPLC purification) of chemically modified pegRNA (cm-pegRNA, featuring 2′-OMe and phosphorothioate modifications and regular length) and of unmodified pegRNA with a 3′-xrRNA motif (equivalent to an IVT xr-pegRNA), against the mouse *Pparg* site for insertion of Kozak motif (Fig. S11A). In two mouse cell lines (LLC and N2a cells), while co-transfection of the cm-pegRNA with plasmid-borne uPEn induced visibly greater levels of accurate edits (than the group with xr-pegRNA), it also undesirably caused higher rates of indels, which eventually translated into sometimes lower edit/indel ratios (Fig. S11B–D). These results suggested that the cm-pegRNA did not necessarily outperform xr-pegRNA (with uPEn) in term of refining the ratio of precise PE/impurities. With the additional consideration of the current high cost of obtaining long, chemically synthesized RNA, our results support the use of IVT, motif-stabilized pegRNA for routine uPEn applications, unless in a context where the absolute editing efficiency is prioritized over editing purity. On another note, while our work was in progress, we noticed a recent study that reported high-efficiency adaptation of nickase-based PE for single nucleotide substitutions in mouse embryos (PE-embyro) (Kim-Yip et al., 2024). Intriguingly, compared to its potencies for point mutations, the PE-embryo method installed larger edits (3–8 bp insertions) with significantly lower efficiencies (Kim-Yip et al., 2024). Therefore, it would be instrumental in future studies to systematically compare uPEn and PE-embryo tools for installing various edit types in animals. Overall, our work strongly supports uPEn as a broadly useful and readily applicable editing platform for livestock genetic improvements and beyond.

## Supplementary Material

pwaf006_suppl_Supplementary_Materials

## Footnotes

We thank Dr. Rihong Guo from the Jiangsu Academy of Agricultural Sciences for invaluable technical assistance. We are grateful to the laboratories of Drs. G Liu, X Gao, Z Lin, and Z Gan for various supports (MARC-NJU). We are thankful to the core facility of MARC-NJU for equipment supports, and to GemPharmatech Inc for services on mouse zygote experiments. We appreciate the help in constructs from Ms. Jingwen Chen (MARC-NJU) and Dr. Yafeng Wang (Gulou Hospital, Nanjing). We thank all members of Wan lab and Liu lab for discussions and assistance.

The authors declare no conflict of interest.

This study was supported by the National Key Research and Development Program of China (NKPs) (2021YFF1000700 and 2019YFA0802802), and by the National Natural Science Foundation of China (32172735).

Y.W., J.L., and Y.Z. designed the project, with the help from F.W. W.M., P.W., L.Z., and D.L. performed and analyzed the experiments. X.L., X.L., and X.H. provided essential technical guidance through prime editing experiments. W.M. made the initial draft of the manuscript. Y.W. and J.L. polished and finalized the manuscript with the help from other authors. Y.W., J.L., F.W., and Y.Z. supervised and coordinated the study.

All institutional and national guidelines for the care and use of laboratory animals were followed.

The targeted deep-sequencing data generated in this study have been submitted to the NCBI BioProject database under accession number PRJNA1095397.
