# Supplementary material for: An upgraded nuclease prime editor platform enables high-efficiency singled or multiplexed knock-in/knockout of genes in mouse and sheep zygotes
Source: Protein Cell. 2025 Jan 20;16(8):732–8. doi: 10.1093/procel/pwaf006 (PMC12342182; doi:10.1093/procel/pwaf006)
Supplement: pwaf006_suppl_Supplementary_Materials [file pwaf006_suppl_supplementary_materials.pdf]

Mao et al. An upgraded nuclease prime editor platform enables high-efficiency singled or multiplexed knock-in/knockout of genes in mouse and sheep zygotes

- **I: Materials and Methods**
- **II: Supplementary Figures (Fig. S1 – Fig. S11)**
- **III: Supplementary Tables 1-6**
- **IV: Key Constructs and Sequences**

I: Materials and Methods:

Ethical statements

The mice experiments were approved by the Institutional Animal Care and Use Committee of Model Animal Research Center of Nanjing University (AP# LJH19). Experiments involving Hu sheep were approved by the Animal Care and Use Committee of Nanjing Agricultural University.

Animals

Animal care and use protocols (mice) were in strict accordance to the Regulation for Management of Laboratory Animals (1988) and Guidelines for Care and Use of Laboratory Animals (2006) issued both by the Ministry of Science and Technology of People's Republic of China. The mice experiments were approved by the Institutional Animal Care and Use Committee of Model Animal Research Center of Nanjing University (AP# LJH19). All mice used were of the C57BL/6JGpt genetic background. Hu sheep were housed at Qidong Ruipeng Animal Husbandry in Jiangsu Province. All protocols involving the use of Hu sheep were performed in accordance with the approved Guidelines for Animal Experiments of Nanjing Agricultural University and were approved by the Animal Care and Use Committee of Nanjing Agricultural University.

Plasmid constructions

The key plasmids in this work are listed in Key construct and sequences section below. The full-length uPEn3 and the nickase-based PEmax plasmids were described previously (Li et al., 2023).

The sequences for PEmax-SPL and PEmax-SPLΔRH, as well as their respective GCN4/scFv forms are reported in the file. The sequences for uPEn and the split/tagged uPEn-ΔRH constructs are also included. For genome targeting, the mouse (Mus musculus) and sheep (Ovis aries) gene sequences are downloaded from NCBI database within the assemblies of GRCm39/mm39 and ARS-UI_Ramb_v3.0, respectively. The *Pparg* regulatory element reporter featured the placement of 699-bp mouse *Pparg*-γ2 sequence (upstream of the start codon) to the 5′-end of enhanced green fluorescent protein (EGFP). The sgRNAs for *MSTN* knockout were selected with the aid of CRISPOR (Concordet and Haeussler, 2018), whereas the pegRNAs were designed to adopt a xr-pegRNA architecture (Zhang et al., 2022). All guide RNA sequences are listed in Supplementary Table 1 below. During vector construction, the Phanta Flash Master Mix (Vazyme) was used for PCR amplification of products greater than 1 kb, and the Phanta Max Master Mix (Vazyme) was used for fragments less than 1 kb. The guide RNA plasmid backbone was amplified from pGL3-U6-sgRNA-EGFP (Addgene #107721). The pegRNA cassette or the sgRNA cassette were first assembled using primers and then cloned into the backbone vector using MultiF Seamless Assembly Mix (ABclonal). The assembly reaction was carried out in a total volume of 20 μl, containing 10 μl of the enzyme mix, together with the longer and shorter fragments (in respective amounts [ng] of 0.02 × number of bases and 0.04 × number of bases).

Cell culture, transfection and fluorescence-based sorting

HEK293T cells and N2a cells were cultured and passaged in Dulbecco's modified Eagle's medium (DMEM) supplemented with 10% (v/v) fetal bovine serum (FBS). The cultures were incubated at 37℃ with 5% CO2. The cells were transfected at 60% confluency with the use of EZ Trans reagent (Life-iLab). For the *Pparg* regulatory element reporter, 0.5 μg of the plasmid (per

well) was transfected in HEK293T cells cultured in 24-well plates. After transfection (48 hours), the cells were harvested and subjected to flow cytometry analysis using the BD LSRFortessa Cell Analyzer. For PEmax-based editing of the reporter, the plasmids were transfected into HEK293T cells (24-well plate, PEmax:pegRNA:reporter = 9:3:1 [total of 650 ng/well]). Each component of the split PEmax (nCas9 and the RT module) was transfected respectively at the same molar amount as the full-length PEmax. After transfection (72 hours), the cells were harvested and subjected to flow cytometry analysis. For gene editing experiments in N2a cells, a total of 1 μg of plasmids (PE:pegRNA at 3:1) was transfected with the use of Lipofectamine 3000 in 24-well plates. 72 hours following transfection, the cells were harvested and subsequently sorted on the positivity of EGFP (encoded by the pegRNA plasmid), using the BD Aria III system. 1×10^4 EGFP-positive cells were collected and samples were prepared for sequencing.

For co-transfection of plasmids and chemically synthesized guide RNA into LLC and N2a cells, Lipofectamine 3000 was utilized. The culture condition of LLC cells was similar to that of HEK293T and N2a cells. The chemically modified pegRNA (cm-pegRNA, featuring 2′-OMe/phosphorothioate modifications and regular length) and of unmodified pegRNA with a 3′-xrRNA motif (equivalent to an IVT xr-pegRNA) were synthesized and HPLC-purified via commercial services (GeneScript). First, 1 μg of DNA (PE plasmids/GFP marker plasmid = 9:1) and 70 μmol of synthesized pegRNA were incubated separately with diluted Lipofectamine 3000. The RNA-containing mix did not contain the P3000 supplement. Following incubation, the mix with DNA and the counterpart with RNA were added together to the cells. Fluorescent sorting of the cells was performed 72 hours post-transfection, based on the EGFP marker.

Genomic DNA extraction and genotyping

Editing at the target sites were first analyzed by Sanger sequencing. Cells were harvested in cell lysis buffer (prepared with dilution of 50 µl 1 M Tris-HCl pH8.0, 25 µl 10%SDS and 200 µl 20 mg/ml Proteinase K into a total volume of 5 ml). The cells were lysed at 37°C for 1 h, followed by incubation at 80°C for 30 min. DNA extraction from animal tissues is carried out with a commercially available kit (TIANGEN). The targeted sites were PCR amplified prior to analyses by Sanger sequencing or next generation sequencing (NGS). The primers used to amplify the target sites are listed in Supplementary Table 2 below. Touchdown PCR protocols were adopted (16 cycles with step reduction of annealing temperature: 65°C to 57°C, at a 0.5°C gradient; and then 20 cycles of regular amplification). The reactions were carried out with 2× Phanta Max Master Mix (Dye Plus) (Vazyme) in 20 µl reaction volume. The PCR products were visualized using 1% agarose gel electrophoresis before submitted to sequencing services.

Western Blot

Cells were lysed in RIPA buffer on ice. The samples were centrifuged at 12,000 revolutions per minute (rpm) at 4°C for 20 minutes. The samples were subjected to SDS-PAGE, followed by immunoblotting. The primary antibodies were purchased from commercial sources (Cas9 [GenScript, A01885], GAPDH [Santa Cruz Biotechnology, sc-32233]). Following primary and secondary antibody incubation, the indicated proteins were detected by chemiluminescence.

In vitro transcription

The template fragments for in vitro transcription were first prepared by PCR to include T7 promoter sequence. The primers involved are presented in Supplementary Table 3 below. Upon validation of the PCR product on 1% agarose gel electrophoresis, the reaction mix was incubated at 37°C for 30 minutes with the Dpn1 enzyme for plasmid removal. Following denaturation at 80°C

for 20 minutes, the remaining DNA fragment underwent purification with the FastPure Gel DNA Extraction Mini Kit from Vazyme (reaction sample clean-up through columns). The actual transcription of mRNA and guide RNAs (gRNA/pegRNA) was conducted using the mMESSAGE mMACHINE T7 and MEGAshortscript T7 kits, respectively, both from Thermo Fisher, according to manufacturer's instructions. In the experiment to prepare the mRNA components of the split/tagged uPEn-ΔRH for mouse editing, a poly(A) tailing step was included to prepare both mRNA modules to potentially aid editor expression (using the mMESSAGE mMACHINE T7 ULTRA kit from Thermo Fisher). For sheep editing with the full-length uPEn or split/tagged uPEn-ΔRH, the in vitro transcription was performed with the mMESSAGE mMACHINE T7 kit without a deliberate poly(A)-tailing step. The in vitro transcribed RNA was purified using the RNA Clean & Concentrator Kit from ZYMO. The final transcription products were then diluted to a standardized concentration of 500 ng/μl, to facilitate later usage. The samples were divided into 5 μl aliquots and stored at -80°C until subsequent experiments.

Editing of animal zygotes

In both mice and sheep, mRNA and guide RNA were delivered by cytoplasmic injection into fertilized eggs, which were then transplanted through the oviduct to the recipient animals. The technical services for mouse zygote injection and transplantation were provided by GemPharmatech Inc. (Nanjing, China), based on similar protocols for Cas9-dependent knockout (Qin et al., 2016). For mouse-editing, 97.5 ng/μl of Cas9-GCN4 and 52.5 ng/μl of scFv-RTaseΔRH mRNA, and 50 ng/μl of pegRNA were delivered via a piezo microinjector into the cytoplasm of 0.5-day fertilized eggs. The fertilized eggs were then transplanted into the pseudo-pregnant females (ICR strain). After pups were delivered, the toe clips of the neonatal mice were collected for genotyping. Subsequently,

approximately at about eight weeks of age, the founder mice were bred with WT C57/BL6 mice to obtain the F1 generation.

For editing of the Hu sheep, healthy ewes with regular estrus cycles were chosen as the zygote donors. The donor sheep were first given a vaginal suppository. Nine days later, superovulation procedure was initiated. After synchronized mating, the one-cell stage embryos were surgically obtained from the donor ewes. The *in vitro* transcribed RNA (100 ng/μl of uPEn [or 65 ng/μl of Cas9-GCN4 and 35 ng/μl of scFv-RTaseΔRH] mRNA, 50 ng/μl of pegRNA and 25 ng/μl of mixed sgRNAs) were microinjected (with a Nikon microinjector) into the cytoplasm of the one-cell embryos. They were cultured in M16 medium (Sigma) at 38.5°C and 5% CO_2 to allow cleavage. The surrogate ewes were prepared in advance. In total, 89 cleaved embryos were transferred into 32 estrous-synchronized recipient sheep. Following about 150 days of gestation, founder lambs were born. The ear cartilage tissue was collected from the newborns for genotyping.

Amplicon deep sequencing and data analysis

The primers used to amplify the target sites are listed in Supplementary Table 2 below. Potential off-target (OT) sites for the pegRNA and sgRNA were predicted using Cas-Offinder and selected at random. The OT sites analyzed in this work are listed in Supplementary Table 4 below. The primers used for OT sites are listed in Supplementary Table 5 below. Barcoded primers were used for PCR amplification of the on-target and off-target sites (~250 bp amplicons). Touchdown PCR protocol was adopted (16 cycles with step reduction of annealing temperature: 65°C to 57°C, at a 0.5°C gradient; and then 25 cycles of regular amplification), and reaction was carried out with 2× Phanta Max Master Mix (Dye Plus) (Vazyme). The pooled samples were purified by the FastPure Gel DNA Extraction Mini Kit (Vazyme, reaction sample clean-up through columns). Sequencing was carried

out on an Illumina HiSeq X Ten platform (NovaSeq-S4-PE150) via the services by Annoroad Gene Technology, Beijing, China. The sequence reads obtained from the deep sequencing were demultiplexed by fastq-multx (Aronesty, 2013), merged via FLASH (Magoč and Salzberg, 2011), and then analyzed using CRISPResso2 (Clement et al., 2019). For *PPARG*-KI analysis, we provided the desirable knock-in allele sequence (in "HDR" mode) to assist the analysis. For *MSTN*-KO analysis (in "NHEJ" mode), we analyzed the knockout efficiency by quantitating frameshifted alleles. For off-target analyses, the NHEJ mode was used on the OT site-derived reads, while an OT-matched sequence was used as the input sgRNA.

Cell isolation, purification and induction of differentiation

The stromal vascular fraction (SVF) was isolated from 10-day-old mice as previously described (Fu et al., 2023). Briefly, for an experiment, the inguinal fat pads were harvested from two homozygous knock-in and two wild-type littermates. The fat pads were enzymatically digested, and the resulting cell suspensions were pooled according to their genotypes. The isolated cells (from 4 mice) were seeded into a total of four wells of a six-well plate, and cultured in the basal medium [high-glucose Dulbecco's Modified Eagle Medium (DMEM) supplemented with 10% fetal bovine serum (FBS) and 1% antibiotic-antimycotic solution (AB/Am)]. Upon confluency, the cells were expanded once in larger vessels. At the next passage, the cells were transferred to experimental plates (12-well). When the cells became confluent, they were subjected to adipogenic induction (*vs.* control culture). A staged differentiation protocol with varying media compositions was employed. The initial phase (days 1-2) featured the supplements of insulin, 3-isobutyl-1-methylxanthine (IBMX), dexamethasone (Dexa), and rosiglitazone in the basal medium. For the next stage (days 3-4), the culture medium was switched to the recipe that contained the supplements of insulin and

rosiglitazone. The final stage (days 5-7) of differentiation was carried out in medium supplemented with rosiglitazone only. After 7 days of induction, cells were harvested for analysis of mRNA and protein expression levels.

Analyses of mRNA levels

Total RNA was isolated using Trizol (Vazyme), followed by reverse transcription to cDNA using a commercial kit with 1 μg RNA as template. Quantitative real-time PCR (qRT-PCR) was performed using AceQ qPCR SYBR Green Master Mix (Vazyme) and gene-specific primers. Amplification was carried out on a Roche LightCycler instrument with the following thermal cycling conditions: initial denaturation at 95°C for 5 min; 40 cycles of 95°C for 10 s and 60°C for 30 s; followed by a melting curve analysis (95°C for 10 s, 65°C for 60 s, and 97°C for 1 s) and a final cooling step at 37°C for 30 s. Primer sequences are provided in Supplementary Table 6 below.

Data analyses

All quantitative readouts with means and deviations were derived from a minimum of three biological replicates. For protein immunoblotting, a representative result from three independent experiments is presented.

II: Supplementary Figures and Legends:

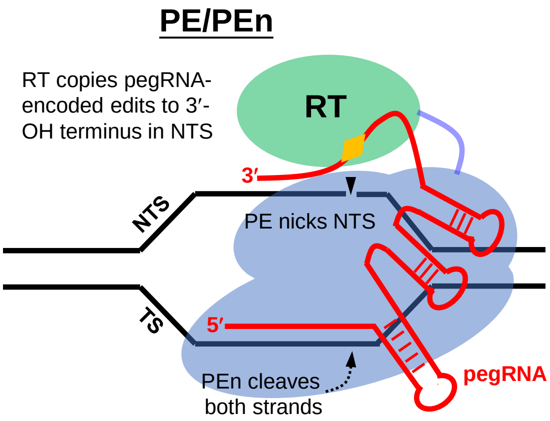

Fig. S1. A schematic overview of the PE/PEn platform. The nCas9 (Cas9) module is depicted in blue, whereas the reverse transcriptase (RT) module is shown in light green. The pegRNA is shown in red, with the portion corresponding to the edit highlighted in yellow. The non-target strand (NTS) and the target strand (TS) at the cleavage site are indicated. The central mechanism for PE/PEn entails RT-dependent reverse transcription of the edit onto the newly formed 3′-OH of NTS, with the 3′ extended sequence of the pegRNA functioning as a template.

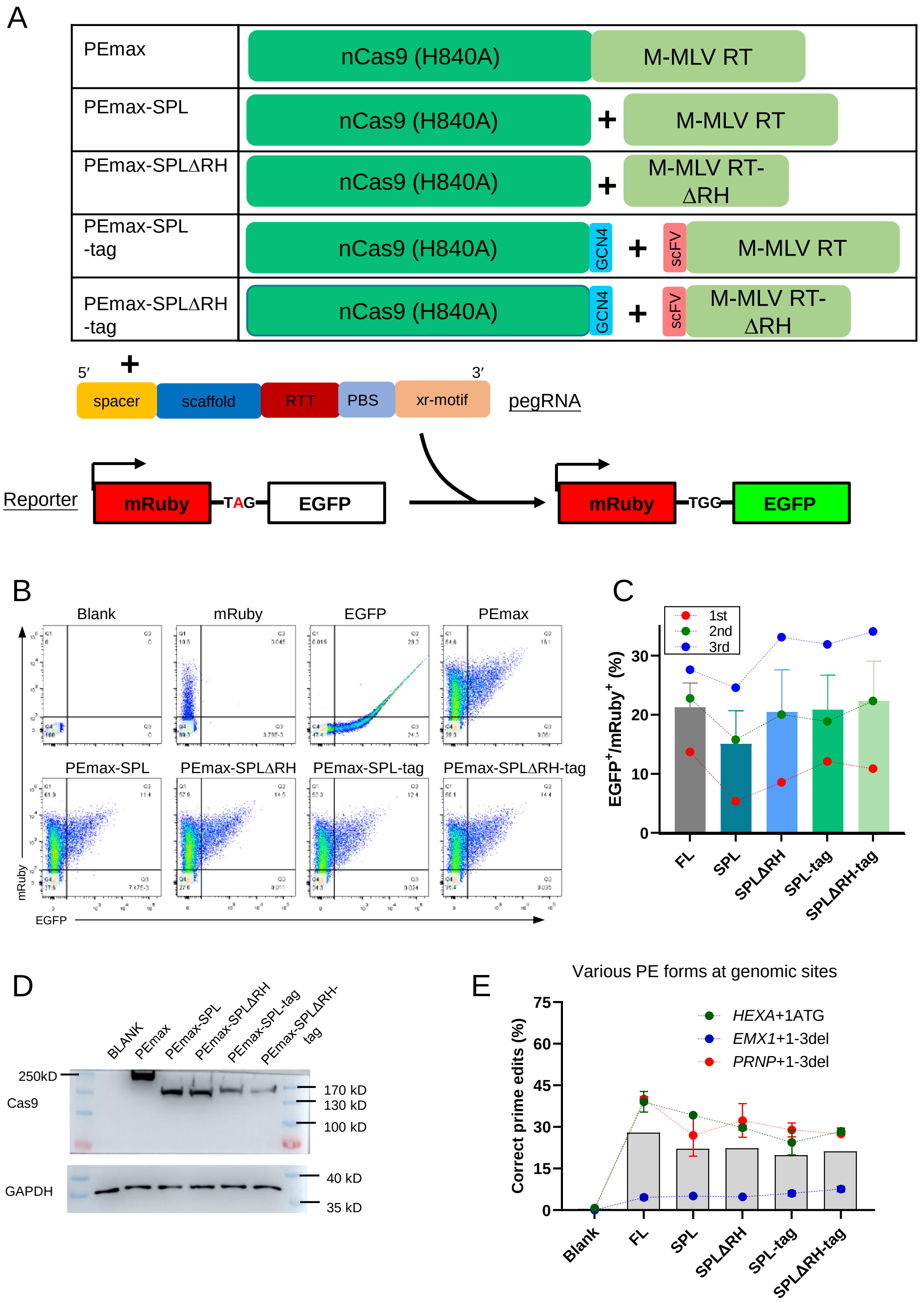

Fig. S2. Validation of the editing efficiencies by different versions of PEmax on a reporter. A. Full-length and different split versions of PEmax constructs are shown in the scheme. For a basic split design, the full-length PEmax was subdivided into separate nCas9 and RTase parts. In another variant split form, the non-essential RNase-H domain was further removed from the RTase (ΔRH).

In parallel, a GCN4 tag (1x) and the corresponding single-chain fragment variable (scFv) binder were respectively placed on the nCas9 and RTase (and the ΔRH) moieties to potentiate their interactions. The pegRNA adopted a design of 3′ xrRNA-joining to enhance editing efficiencies. The PE efficiencies would be indicated by their activities to disable a 'premature' TAG stop codon in a reporter that prevents the translation of a fused EGFP moiety. **B.** Representative results of flow cytometry are shown. The relative levels of EGFP positivity indicate the editing efficiencies for each PE variant. **C.** The editing efficiencies are quantitatively determined using the Q2/(Q1+Q2) formula (±SEM). Individual data points from three independent experiments are marked on the quantitation bars. **D.** The expression levels of transfected PEs for editing were analyzed by Western Blotting with the antibody against Cas9. Data and error bars in this figure show the mean ± SEM. of three biological replicates (n = 3). **E.** The editing of human genomic loci by different versions of the editors on HEK293T cells. Individual data points are derived from NGS analyses of different loci (n = 3 biological replicates, ±SD). The quantitation bars represent the mean editing efficiencies at the 3 tested sites.

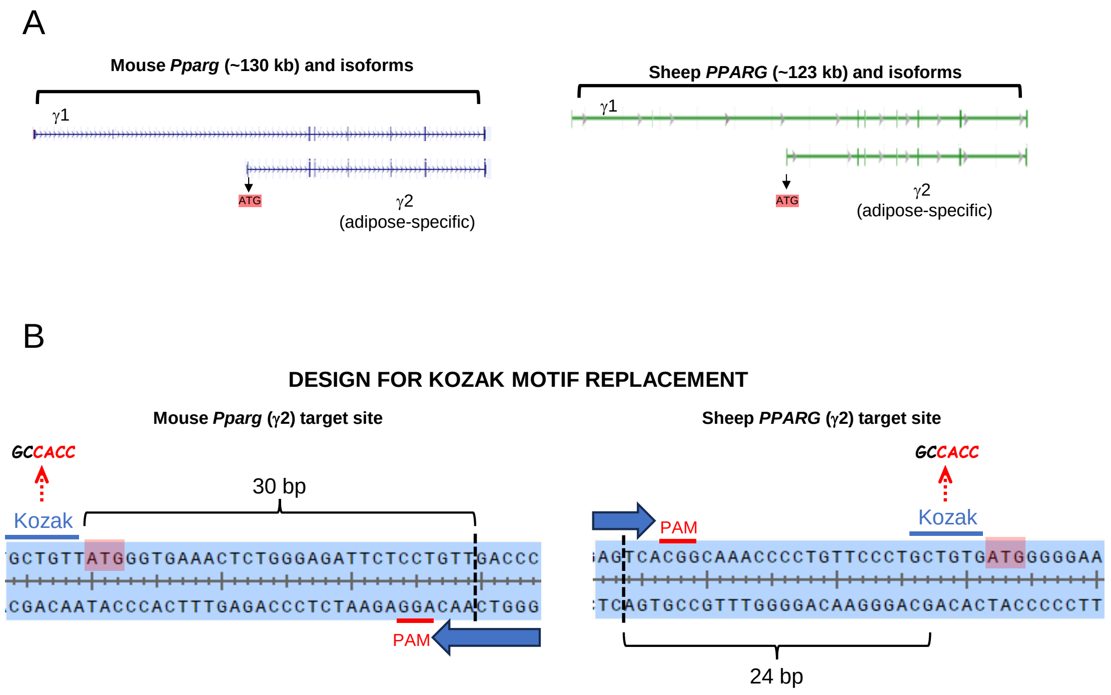

Fig. S3. Potential installation of a consensus Kozak motif to replace the original ones in mouse and sheep *PPARG*. A. The maps indicate mouse and sheep *PPARG* loci. The adipose-specific γ2 isoforms and the corresponding start codons are marked. **B.** The pegRNAs could be designed for this purpose to replace the original Kozak sequence at the mouse and sheep *PPARG* (γ2) sites into the consensus of "GCCACC". As sequence replacement by PE would require a cleavage position upstream of the intended changes, the available PAM and the according cleavage position (as indicated by the dotted line) are located relatively distant from the Kozak sequence in both mouse and sheep *PPARG* sites.

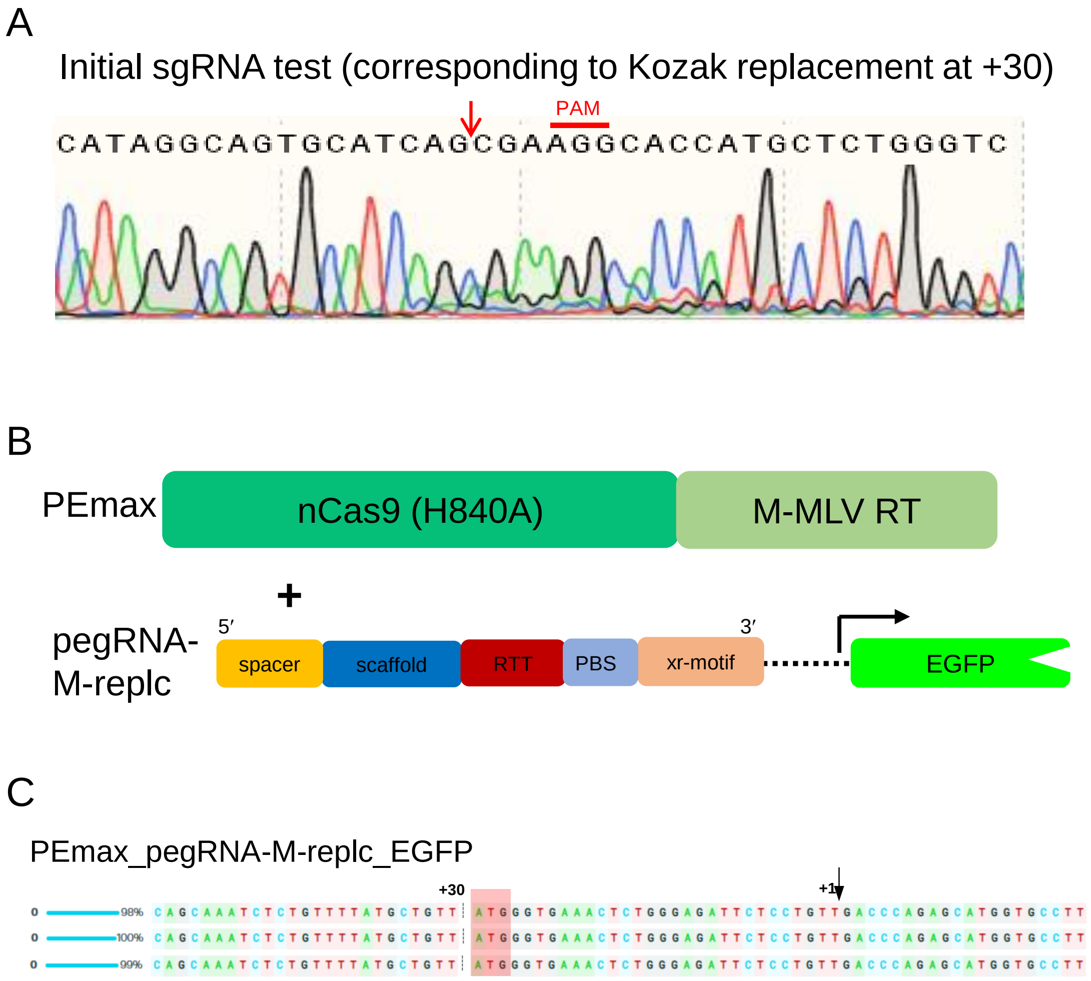

Fig. S4. The Kozak motif replacement in mouse *Pparg* by PEmax appeared challenging. A. An sgRNA corresponding to the pegRNA for replacement of mouse *Pparg* Kozak motif was transfected with Cas9 into mouse N2a cells. Sanger sequencing of amplicons from the target site shows evident cleavage activity. **B.** Subsequently, PEmax and the corresponding pegRNA were co-transfected to N2a cells. The plasmid constructs are illustrated. **C.** Samples from PEmax/pegRNA-transfected cells were subjected to Sanger sequencing. The results were further analyzed by ICE tool (Synthego, (Hsiau et al., 2019)), which showed minimal editing at the Kozak motif.

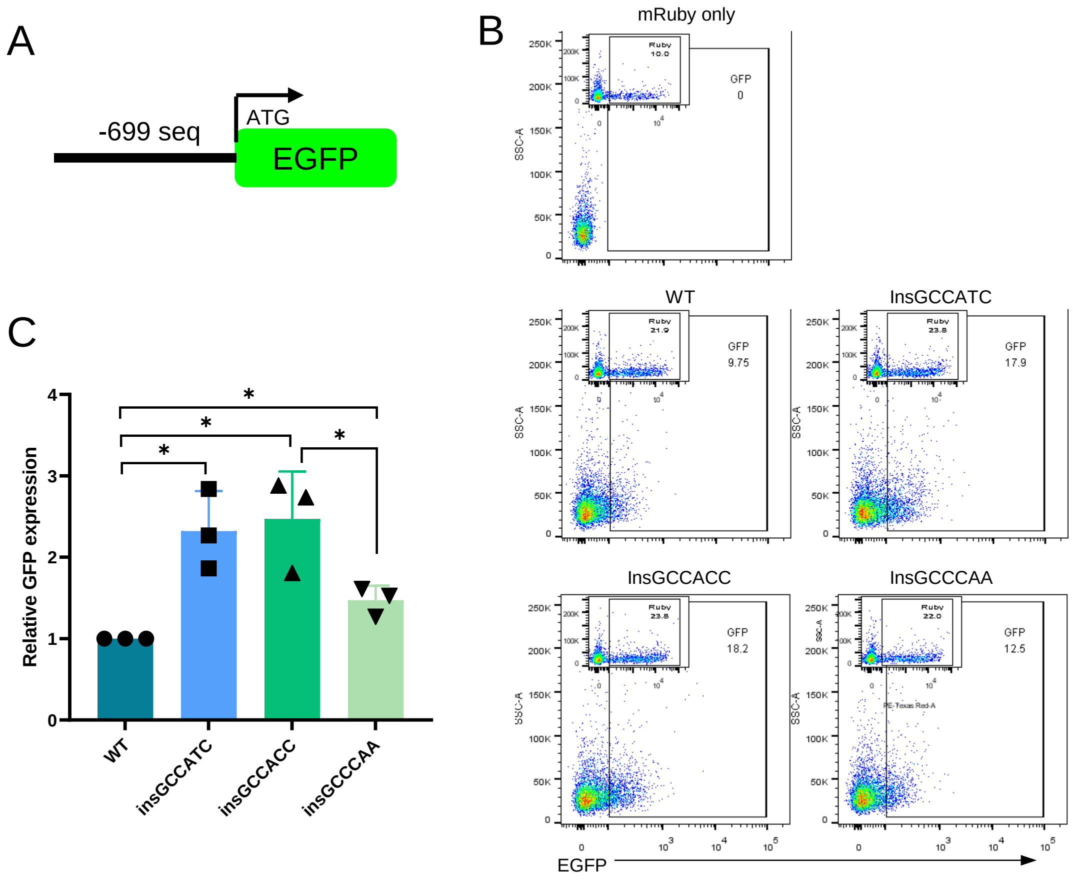

Fig. S5. Direct insertion of Kozak motif may represent an alternative to potentially enhance *Pparg* expression. A. A mouse *Pparg* (γ2) upstream sequence-dependent reporter is illustrated. A 699-bp sequence upstream of the ATG of *Pparg* (γ2) was used to drive the expression of EGFP. **B, C.** HEK293T cells were co-transfected with each *Pparg* (γ2) upstream element-reporter (with different Kozak sequences) and a marker plasmid (CMV-mRuby). Representative results (of 3 independent experiments) of mRuby gating and the subsequent measurement of EGFP fluorescence are shown in (B). The relative levels (normalized to the WT reporter level in each experiment) of EGFP fluorescence from samples of various Kozak sequence insertions are summarized (C). Data and error bars show the mean ± SD from 3 independent experiments. One sample t-tests were performed for comparisons of modified Kozak groups with the WT reporter. For comparisons between the "GCCACC" group and the "GCCCAA" group, unpaired t-test was performed (*P <0.05).

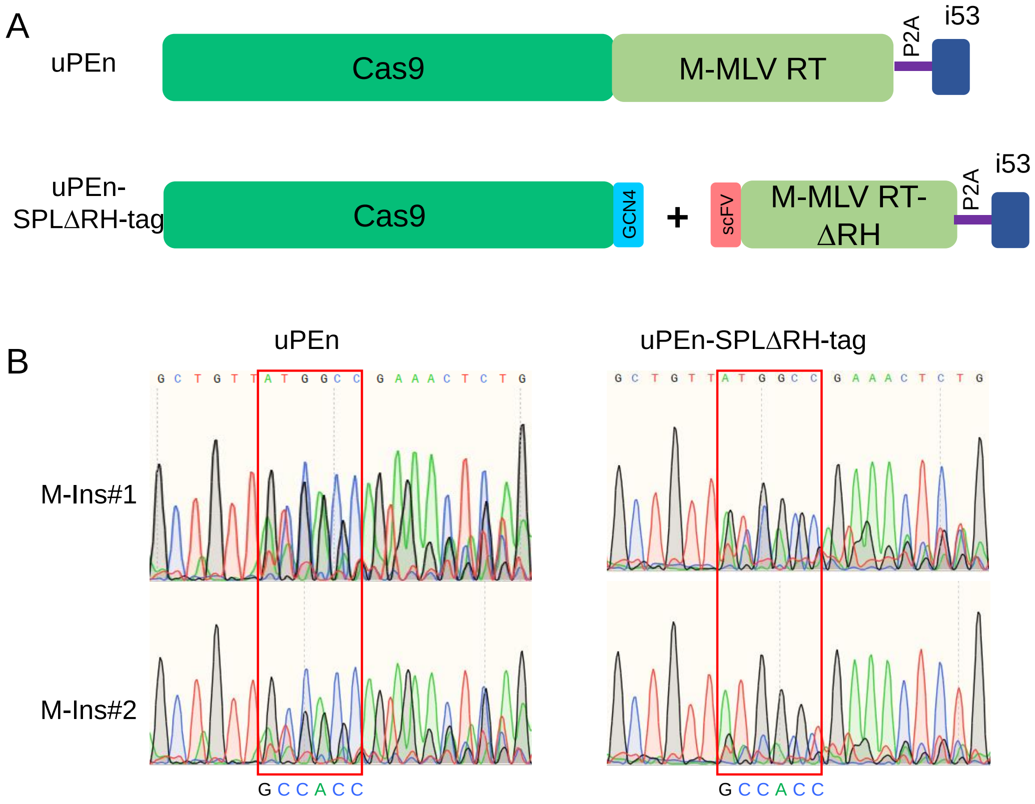

Fig. S6. Evaluation of uPEn-mediated Kozak sequence insertions in N2a cells. A. The constructs for uPEn and uPEn-SPLΔRH-tag are illustrated. The i53 module is depicted in dark blue color. **B.** The N2a cells were co-transfected with uPEn or uPEn-SPLΔRH-tag construct(s), together with the indicated pegRNAs (see Fig. 1B-D). Cells were harvested 72 h after transfection. Sanger sequencing chromatograms provide evidence of significant levels of correct insertion in all groups.

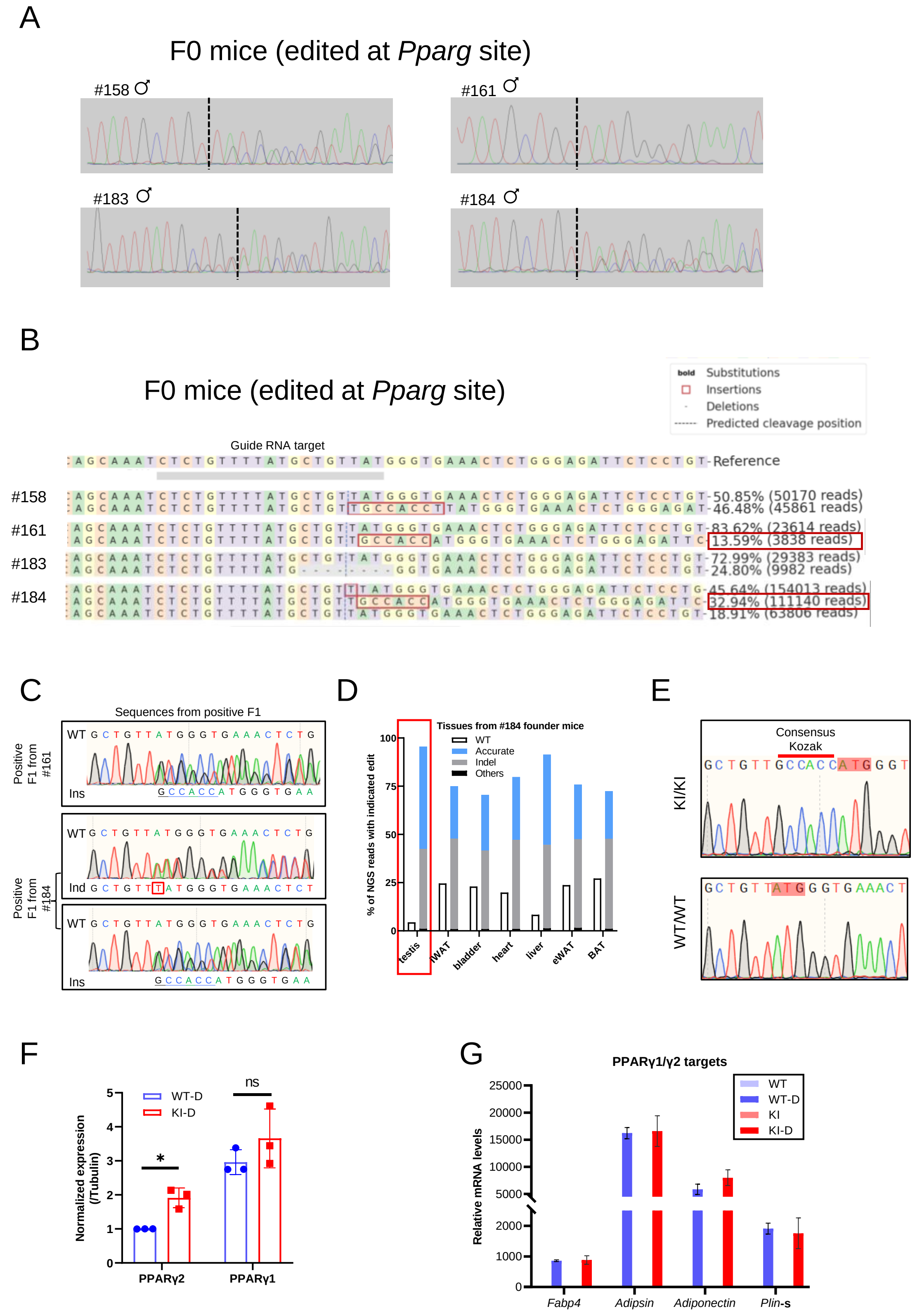

Fig. S7. Genotypes of *Pparg* (γ2)-modified mice and initial characterization of adipocytes derived from the mice. A. Sanger sequencing results of the founder mice-derived samples that indicate any editing at the *Pparg* (γ2) site are shown. The site of cleavage is marked by a black dotted line. **B.** Next-generation sequencing of the target site was carried out on the same samples

from the edited founder mice. The reads were mapped by the use of CRISPResso2. Different allele sequences within each sample are shown. #161 and #184 founders harbored precisely edited alleles. **C.** The founder mice were bred with WT mice to obtain the F1 generation. The toe clips were subjected to Sanger sequencing. The F1 mice showed either the WT/WT or heterozygous WT/edited genotypes. The chromatograms corresponding to the WT/edited genotypes from the initial 1~2 litters of #161 and #184 F1 mice are displayed. The desirable genotype (with correct GCCACC insertion) was observed in F1 mice of both lines. Note that among the progenies of #184 founder, some with the undesirable indel edit was also identified. **D.** We sacrificed the aged #184 founder to harvest tissue samples from testis and several other internal organs. The samples were subjected to targeted NGS. The low representation of the WT allele in the testis tissue is noted (red box). **E.** Genotyping results from a homozygous knock-in (KI/KI) mouse and one of its WT littermates (F2 generation). **F**. The SVF cells isolated from the subcutaneous inguinal adipose tissue of the WT and the homozygous knock-in (KI) mice were subjected to in vitro adipogenic induction. Each genotype/condition was represented by three replicate wells. On day-7 following induction, the protein samples were harvested. Following IB analyses, the relative levels (± SD) of PPARγ1 and PPARγ2 in differentiated cells (WT-D and KI-D) were quantitated in reference to those of Tubulin. Quantitation was based on averaged values from 3 independent experiments (each with 2~3 biological replicates). Two-sided student's t-tests were used to determine the statistical differences between genotypes (*: $P<0.05$, ns: not significant). **G.** PPARγ downstream target gene expression (normalized to 36B4) in control and differentiated ("D") cells from the WT and KI/KI mice. Data are presented as mean ± SEM (n = 3 biological replicates).

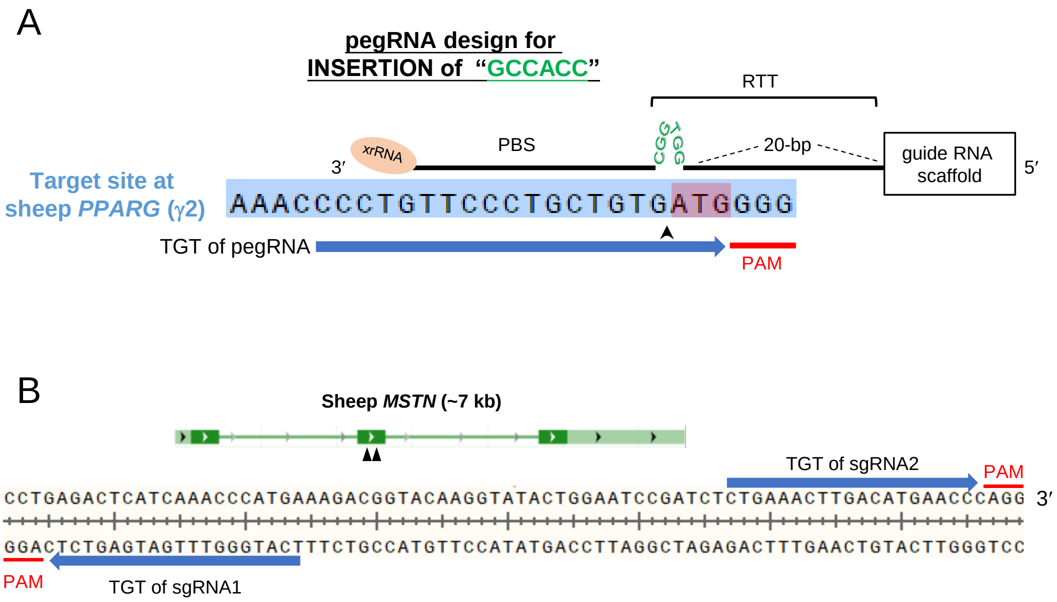

Fig. S8. Designs of pegRNA and sgRNA for editing the Hu sheep. A. Design of the pegRNA to insert a consensus Kozak motif upstream of sheep *PPARG* is illustrated. The PAM (red), the guide RNA-complementary sequence (dark blue), and the cleavage point (black arrowhead) are marked. The PBS and RTT for the pegRNA is also schematically shown on top of the target sequence. The sequence in green corresponds to the consensus Kozak motif to be inserted directly upstream of the start codon. **B.** The positions of the two sgRNAs targeting the second exon of *MSTN* gene are shown in the scheme.

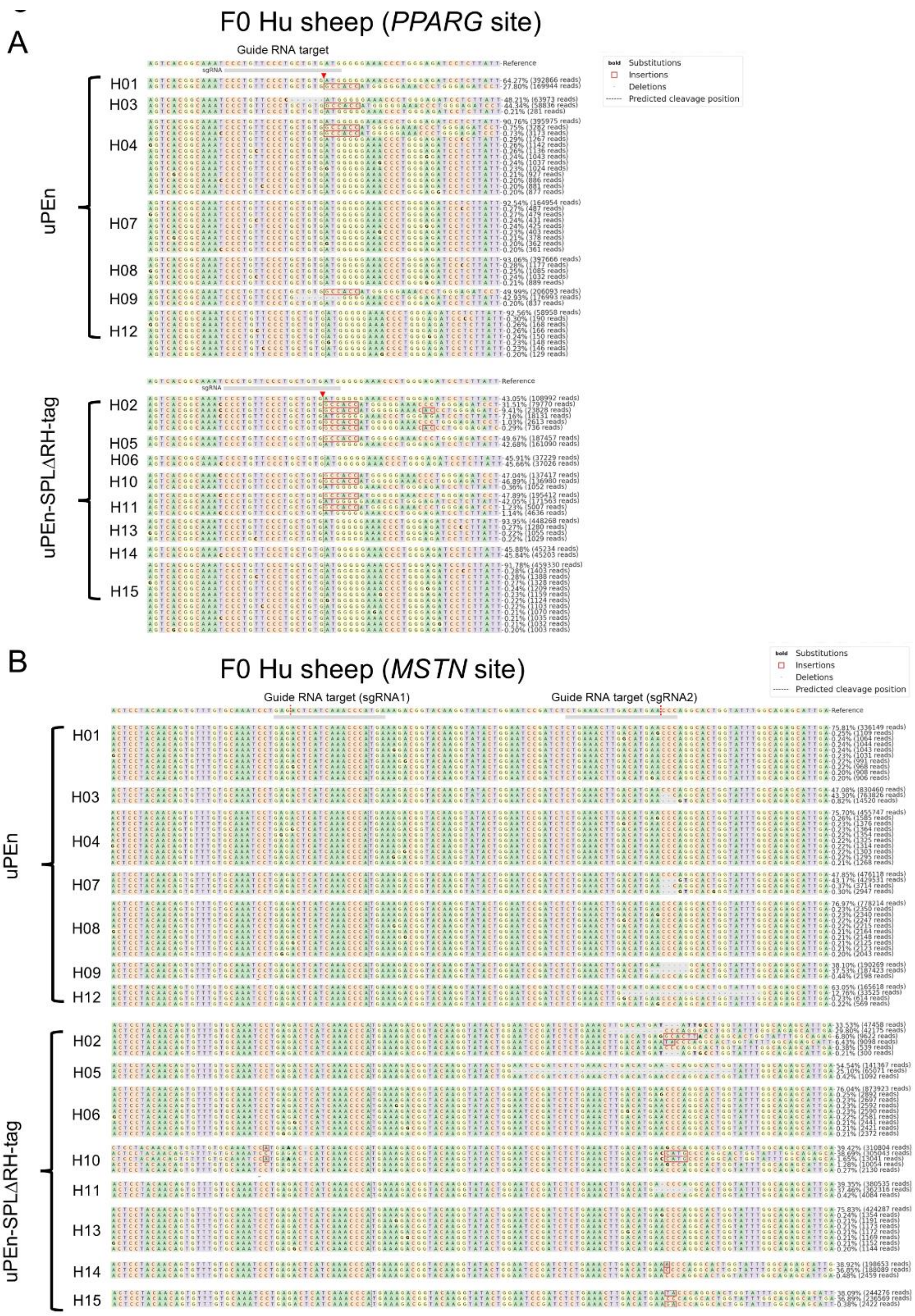

Fig. S9. Determination of knock-in and knockout efficiencies in Hu sheep. A, B. The Hu sheep zygotes were microinjected with a mixture of uPEn or uPEn-SPLΔRH-tag mRNA, a pegRNA (*PPARG*) and two sgRNAs (*MSTN*). The cleaved embryos were transplanted to surrogate sheep. Fifteen experimental lambs were born after a period of ~ 150 days. Next generation sequencing of the *PPARG* (A) and *MSTN* (B) sites was carried out on samples from all the newborn lambs. The

reads were mapped by the use of CRISPResso2. Different allele sequences within each sample are shown.

Off-Target analyses for the pegRNA and sgRNA2

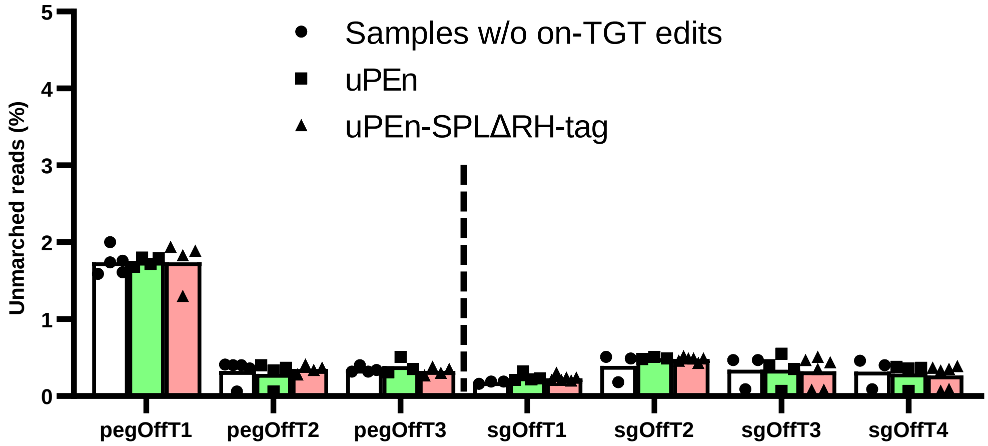

Fig. S10. Analysis of uPEn-associated off-target effects upon sheep editing. Potential off-target sites corresponding to the pegRNA (left of the dotted line) and to the sgRNA2 (right) were predicted by the use of Cas-OFFinder (mismatched bases limited to five). Several sites were picked at random for the pegRNA (3) and sgRNA2 (4). The potential off-target sites were amplified from samples of edited newborns. Samples from three newborns with no on-target edits were also used for amplification in parallel. The reads were mapped by the use of CRISPResso2. Each OT site is shown separately. For comparisons, the percent of unmatched reads (of total reads) in the edit-positive uPEn and uPEn-SPLΔRH-tag samples were displayed alongside those in the edit-negative samples.

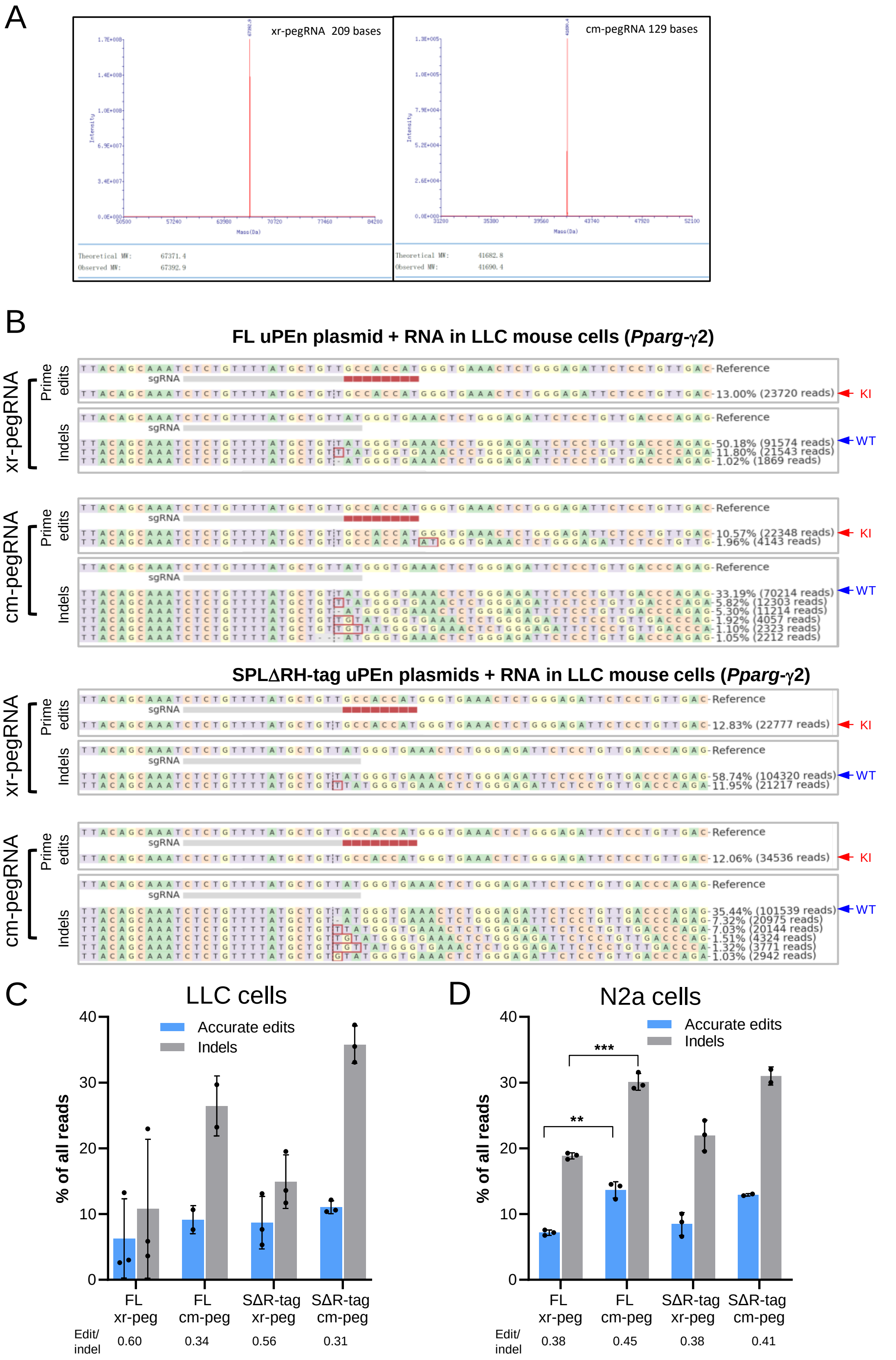

Fig. S11. Comparison of the efficiencies of chemically modified pegRNA and 3′ xrRNA motif-adjoined pegRNA. Chemically modified pegRNA (cm-pegRNA) and non-chemically modified

pegRNA (xr-pegRNA) were obtained through commercial services. The cm-pegRNA (regular length) was modified with 2′-OMe and phosphorothioate linkages at three positions at the 5′ and 3′ termini. The xr-pegRNA was not chemically modified, but contained the xrRNA-motif at the 3′ end. **A.** The Electrospray Ionization (ESI) Mass Spectrometry results of the two oligo-RNAs are provided. **B, C.** The two pegRNAs (in RNA format), together with plasmid-borne uPEn were co-transfected for editing the genomic locus in mouse LLC cells. An EGFP-encoding plasmid was also included in the transfection mix. 72 h post-transfection, the $EGFP^+$ cells were harvested by FACS sorting. The samples were subjected to NGS. Representative allele frequency graphs are shown in (**B**), while the quantifications of accurate edits and indels are presented in (**C**). Except for a few quantitation bars (representing 2 biological replicates), most of the data are summarized from 3 biological replicates (mean ± SD). **D.** The mouse N2A cells were transfected with the pegRNAs and plasmids as in (B). The quantifications of accurate edits and indels are presented. Except for a few quantitation bars (representing 2 biological replicates), most of the data are summarized from 3 biological replicates (mean ± SD). Student's t-test was performed for comparisons of accurate edits and indels between the xr-pegRNA and cm-pegRNA groups (with FL-uPEn) (*: $P<0.05$).

III: Supplementary Tables:

Supplementary Table 1 - Guide RNA sequences

Species	NO.	gene	guide RNA target sequence	Sequence for homology, edits , PBS and xrRNA
Mus musculus	pegRNA-M-replc	Pparg	GCACCATGCTCTGGGTCAAC	AGCAAATCTCTGTTTTATGCCACCATGGGTGAAACTCTGGGAGATTCTCCTGTTGACCCAGAGCATGTGTCAGGCCTGCTAGTCAGCCACAGTTTGGGGAAAGCTGTGCAGCCTGTAACCCCCCCAGGAGAAGCTGGGAAACCAAGC
	pegRNA-M-Ins#1	Pparg	CTCTGTTTTATGCTGTTAT	AGAGTTTCACCCATGGTGGCAACAGCATAAAACATGTCAGGCCTGCTAGTCAGCCACAGTTTGGGGAAAGCTGTGCAGCCTGTAACCCCCCCAGGAGAAGCTGGGAAACCAAGC
	pegRNA-M-Ins#2	Pparg	TCTCTGTTTTATGCTGTTA	AGAGTTTCACCCATGGTGGCAACAGCATAAAACAGTGTCAGGCCTGCTAGTCAGCCACAGTTTGGGGAAAGCTGTGCAGCCTGTAACCCCCCCAGGAGAAGCTGGGAAACCAAGC
Ovis_aries	sgRNA1	MSTN	TCATGGGTTTGATGAGTCTC	/
	sgRNA2	MSTN	CTGAAACTTGACATGAACCC	/
	pegRNA-S-Ins	PPARG	CCCTGTTCCCTGCTGTGATG	TCTCCCAGGGTTTCCCCCATGGTGGCCACAGCAGGGAACAGTGTCAGGCCTGCTAGTCAGCCACAGTTTGGGGAAAGCTGTGCAGCCTGTAACCCCCCCAGGAGAAGCTGGGAAACCAAGCT

Supplementary Table 2 - Primers for amplification of the target sites

NO.	Barcode	F	R	Species
m-pparg-peg-1	CTGCTGAA	CTGCTGAACATAAGCTCGATGACCATAAGCC	GTGCCCGAAGCATCCCTTGCAGCAACAT	Mouse
m-pparg-peg-2	GAAGCTGT	GAAGCTGTCATAAGCTCGATGACCATAAGCC	GTGCCCGAAGCATCCCTTGCAGCAACAT	Mouse
m-pparg-peg-3	TCGTTCAT	TCGTTCATCATAAGCTCGATGACCATAAGCC	GTGCCCGAAGCATCCCTTGCAGCAACAT	Mouse
m-pparg-peg-4	CCATAGTT	CCATAGTTCATAAGCTCGATGACCATAAGCC	GTGCCCGAAGCATCCCTTGCAGCAACAT	Mouse
s-pparg-peg-1	CTACAG	CTACAGGGGTTCCCAAGTTTTACTGCC	GGTACTTGCAGACACGGTGA	Sheep
s-pparg-peg-2	TGACTC	TGACTCGGGTTCCCAAGTTTTACTGCC	GGTACTTGCAGACACGGTGA	Sheep
s-pparg-peg-3	TCTTGC	TCTTGCGGGTTCCCAAGTTTTACTGCC	GGTACTTGCAGACACGGTGA	Sheep
s-pparg-peg-4	ATACGG	ATACGGGGGTTCCCAAGTTTTACTGCC	GGTACTTGCAGACACGGTGA	Sheep
s-pparg-peg-5	AACACG	AACACGGGGTTCCCAAGTTTTACTGCC	GGTACTTGCAGACACGGTGA	Sheep
s-pparg-peg-6	TAACCG	TAACCGGGGTTCCCAAGTTTTACTGCC	GGTACTTGCAGACACGGTGA	Sheep
s-pparg-peg-7	TGTTCG	TGTTCGGGGTTCCCAAGTTTTACTGCC	GGTACTTGCAGACACGGTGA	Sheep
s-pparg-peg-8	CATGTA	CATGTAGGGTTCCCAAGTTTTACTGCC	GGTACTTGCAGACACGGTGA	Sheep
s-pparg-peg-9	ATGTCT	ATGTCTGGGTTCCCAAGTTTTACTGCC	GGTACTTGCAGACACGGTGA	Sheep
s-pparg-peg-10	GATACG	GATACGGGGTTCCCAAGTTTTACTGCC	GGTACTTGCAGACACGGTGA	Sheep
s-pparg-peg-11	CACATG	CACATGGGGTTCCCAAGTTTTACTGCC	GGTACTTGCAGACACGGTGA	Sheep
s-pparg-peg-12	TTGTGA	TTGTGAGGGTTCCCAAGTTTTACTGCC	GGTACTTGCAGACACGGTGA	Sheep
s-pparg-peg-13	AGTCAG	AGTCAGGGGTTCCCAAGTTTTACTGCC	GGTACTTGCAGACACGGTGA	Sheep
s-pparg-peg-14	GAGTTG	GAGTTGGGGTTCCCAAGTTTTACTGCC	GGTACTTGCAGACACGGTGA	Sheep
s-pparg-peg-15	AGACTA	AGACTAGGGTTCCCAAGTTTTACTGCC	GGTACTTGCAGACACGGTGA	Sheep
s-pparg-dsg-1	GATGAG	GATGAGCACAATAAAGTAGTAAAGGCCC	GCCTAAGTTGGATTCAGGTTGT	Sheep
s-pparg-dsg-2	TAAGGT	TAAGGTCACAATAAAGTAGTAAAGGCCC	GCCTAAGTTGGATTCAGGTTGT	Sheep
s-pparg-dsg-3	AAACCG	AAACCGCACAATAAAGTAGTAAAGGCCC	GCCTAAGTTGGATTCAGGTTGT	Sheep
s-pparg-dsg-4	GTGAAT	GTGAATCACAATAAAGTAGTAAAGGCCC	GCCTAAGTTGGATTCAGGTTGT	Sheep
s-pparg-dsg-5	CAGTAG	CAGTAGCACAATAAAGTAGTAAAGGCCC	GCCTAAGTTGGATTCAGGTTGT	Sheep
s-pparg-dsg-6	ATCCAG	ATCCAGCACAATAAAGTAGTAAAGGCCC	GCCTAAGTTGGATTCAGGTTGT	Sheep
s-pparg-dsg-7	TCTTCG	TCTTCGCACAATAAAGTAGTAAAGGCCC	GCCTAAGTTGGATTCAGGTTGT	Sheep
s-pparg-dsg-8	CTTTCA	CTTTCACACAATAAAGTAGTAAAGGCCC	GCCTAAGTTGGATTCAGGTTGT	Sheep
s-pparg-dsg-9	GTAACG	GTAACGCACAATAAAGTAGTAAAGGCCC	GCCTAAGTTGGATTCAGGTTGT	Sheep
s-pparg-dsg-10	CTTAGC	CTTAGCCACAATAAAGTAGTAAAGGCCC	GCCTAAGTTGGATTCAGGTTGT	Sheep
s-pparg-dsg-11	CTGTAA	CTGTAACACAATAAAGTAGTAAAGGCCC	GCCTAAGTTGGATTCAGGTTGT	Sheep
s-pparg-dsg-12	GCAAAG	GCAAAGCACAATAAAGTAGTAAAGGCCC	GCCTAAGTTGGATTCAGGTTGT	Sheep
s-pparg-dsg-13	GCTAAT	GCTAATCACAATAAAGTAGTAAAGGCCC	GCCTAAGTTGGATTCAGGTTGT	Sheep
s-pparg-dsg-14	TACCTT	TACCTTCACAATAAAGTAGTAAAGGCCC	GCCTAAGTTGGATTCAGGTTGT	Sheep
s-pparg-dsg-15	CGTTTG	CGTTTGCACAATAAAGTAGTAAAGGCCC	GCCTAAGTTGGATTCAGGTTGT	Sheep

Supplementary Table 3 - Primers for in vitro transcription

NO.	F		R	
MSTN-sgRNA1	MSTN-sgRNA1-TF	AATTCTTTAAAAAAAAGCTTGGTTTCCCAGCTTCTCCTGGGGGGGTTACAGG	MSTN-sgRNA-TR	AAAAGCACCGACTCGGTGCCAC
MSTN-sgRNA2	MSTN-sgRNA2-TF	TAATACGACTCACTATAGCTGAAACTTGACATGAACCCGTTTTAGAGCTAGAA	MSTN-sgRNA-TR	AAAAGCACCGACTCGGTGCCAC
PPARG-MpegRNA	PPARγ-MpegRNA-TF	TAATACGACTCACTATAGCTCTGTTTTATGCTGTTATGTTTCAGAGCTAGAAAT	PPARγ-MpegRNA-TR	AAAAAGCTTGGTTTCCCAGCTTC
PPARG-SpegRNA	PPARγ-SpegRNA-TF	TAATACGACTCACTATAGCCCTGTTCCCTGCTGTGATGGTTTCAGAGCTAGAAATAGC	PPARγ-SpegRNA-TR	AAAAAGCTTGGTTTCCCAGCTTC
uPEn mRNA	uPEn-TF	TAATACGACTCACTATAGGGAGAGCCGCCACCATGAAACGG	uPEn-TR	TGGTTCTTTCCGCCTCAGAAGCC
Cas9-GCN4	uPEn-TF	TAATACGACTCACTATAGGGAGAGCCGCCACCATGAAACGG	uPEn-TR	TGGTTCTTTCCGCCTCAGAAGCC
scFv-RTase	uPEn-TF	TAATACGACTCACTATAGGGAGAGCCGCCACCATGAAACGG	uPEn-TR	TGGTTCTTTCCGCCTCAGAAGCC
scFv-RTaseDRH	uPEn-TF	TAATACGACTCACTATAGGGAGAGCCGCCACCATGAAACGG	uPEn-TR	TGGTTCTTTCCGCCTCAGAAGCC

Supplementary Table 4 - OT sites analyzed

NO.	Chr.	Off-target site	Mis-matches
PPARG2-peg-OT1	Chr1	CCCTGTcCCCaGCTcTGcTG	4
PPARG2-peg-OT2	Chr2	tCCTGTTCCCTGCTGaccTG	4
PPARG2-peg-OT3	Chr9	CtCTGcTCCCTGCTGgGgTG	4
MSTN-sg2-OT1	Chr3	tTGgAACTTGgCATaAACCC	4
MSTN-sg2-OT2	Chr10	CTGAAtCTgGACATtAcCCC	4
MSTN-sg2-OT3	Chr4	CaGAAACTTcACAgGAACCC	3
MSTN-sg2-OT4	Chr2	CTGAAAtggGACcTGAACtC	5

Supplementary Table 5 - Primers for amplification of OT sites

NO.	Primer-F (5'→3')		Primer-R (5'→3')	
PPARG2-peg-OT1	PPARG2-peg-OT1-F	AGAGTCTGCTGAGGGAGACTGAG	PPARG2-peg-OT1-R	GGCATCCTGCTGTTTTCGGTCGT
PPARG2-peg-OT2	PPARG2-peg-OT2-F	TGAGAGACTCATTTCTGGCCTCA	PPARG2-peg-OT2-R	CAGGGAGAAAGTTCGGAGGAAAA
PPARG2-peg-OT3	PPARG2-peg-OT3-F	CCCACTCTGGCTCATCAAGCTCT	PPARG2-peg-OT3-R	AGCCATGGCGCTAGCTCCCTTCT
MSTN-sg2-OT1	MSTN-dsg2-OT1-F	TTCTGCTAGGCACAAGAAATCAC	MSTN-dsg2-OT1-R	TCTGATGTCTTGATAACTGGCAC
MSTN-sg2-OT2	MSTN-dsg2-OT2-F	CTAGCACACAGAGTAGAAGCCAC	MSTN-dsg2-OT2-R	AAGACCCTATGCTAGGATTTCTC
MSTN-sg2-OT3	MSTN-dsg2-OT3-F	GAGAAAATCACAAATGGTCCCCC	MSTN-dsg2-OT3-R	GCTCTGACTTTCAAAAGTCTTCC
MSTN-sg2-OT4	MSTN-dsg2-OT4-F	GCCATCCTGATTGTTCTTTGT	MSTN-dsg2-OT4-R	ATTGGGGCATCTGAGTGAATT

Supplementary Table 6 - Primers of qRT-PCR

Gene	Primer-F (5'→3')	Primer-R (5'→3')
Pparg (γ1)	AACAGCCTGACGGGGTCTCG	GTCCTGAATATCAGTGGTTCACCGC
Pparg (γ2)	GCTCCACACTATGAAGACATTCCATTCAC	TTTTCAGAATAATAAGGTGGAGATGCAGGT
36B4	GCTTCGTGTTCACCAAGGAGGA	GTCCTAGACCAGTGTTCTGAGC
aP2 (*Fabp4*)	ACACCGAGATTTCCTTCAAACTG	CCATCTAGGGTTATGATGCTCTTCA
Adipsin	CATGCTCGGCCCTACATGG	CACAGAGTCGTCATCCGTCAC
Adiponecti n	GCACTGGCAAGTTCTACTGCAA	GTAGGTGAAGAGAACGGCCTTGT
Perilipin	GGCCTGGACGACAAAACC	CAGGATGGGCTCCATGAC

IV: Key Constructs and Sequences:

PEmax (nCas9-RT):

atgaaacggacagccgacggaagcgagttcgagtcaccaaagaagaagcggaaagtcgacaagaagtacagcatcggcctggacatcggcaccaactctgtgggctgggccgtgatcaccg
acgagtacaaggtgcccagcaagaaattcaaggtgctgggcaacaccgaccggcacagcatcaagaagaacctgatcggagccctgctgttcgacagcggcgaaacagccgaggccacccg
gctgaagagaaccgccagaagaagatacaccagacggaagaaccggatctgctatctgcaagagatcttcagcaacgagatggccaaggtggacgacagcttcttccacagactggaagagt
ccttcctggtggaagaggataagaagcacgagcggcaccccatcttcggcaacatcgtggacgaggtggcctaccacgagaagtaccccaccatctaccacctgagaaagaaactggtggaca
gcaccgacaaggccgacctgcggctgatctatctggccctggcccacatgatcaagttccggggccacttcctgatcgagggcgacctgaaccccgacaacagcgacgtggacaagctgttcatc
cagctggtgcagacctacaaccagctgttcgaggaaaaccccatcaacgccagcggcgtggacgccaaggccatcctgtctgccagactgagcaagagcagaaagctggaaaatctgatcgcc
cagctgcccggcgagaagaagaatggcctgttcggaaacctgattgccctgagcctgggcctgacccccaacttcaagagcaacttcgacctggccgaggatgccaaactgcagctgagcaag
gacacctacgacgacgacctggacaacctgctggcccagatcggcgaccagtacgccgacctgtttctggccgccaagaacctgtccgacgccatcctgctgagcgacatcctgagagtgaacac
cgagatcaccaaggcccccctgagcgcctctatgatcaagagatacgacgagcaccaccaggacctgaccctgctgaaagctctcgtgcggcagcagctgcctgagaagtacaaagagattttct
tcgaccagagcaagaacggctacgccggctacattgacggcggagccagccaggaagagttctacaagttcatcaagcccatcctggaaaagatggacggcaccgaggaactgctcgtgaag
ctgaagagagaggacctgctgcggaagcagcggaccttcgacaacggcagcatcccccaccagatccacctgggagagctgcacgccattctgcggcggcaggaagatttttacccattcctga
aggacaaccgggaaaagatcgagaagatcctgaccttccgcatcccctactacgtgggccctctggccaggggaaacagcagattcgcctggatgaccagaaagagcgaggaaaccatcaccc
cctggaacttcgaggaagtggtggacaagggcgcttccgcccagagcttcatcgagcggatgaccaacttcgataagaacctgcccaacgagaaggtgctgcccaagcacagcctgctgtacg
agtacttcaccgtgtataacgagctgaccaaagtgaaatacgtgaccgagggaatgagaaagcccgccttcctgagcggcgagcagaaaaaggccatcgtggacctgctgttcaagaccaacc
ggaaagtgaccgtgaagcagctgaaagaggactacttcaagaaaatcgagtgcttcgactccgtggaaatctccggcgtggaagatcggttcaacgcctccctgggcacataccacgatctgctg
aaaattatcaaggacaaggacttcctggacaatgaggaaaacgaggacattctggaagatatcgtgctgaccctgacactgtttgaggacagagagatgatcgaggaacggctgaaaacctatg
cccacctgttcgacgacaaagtgatgaagcagctgaagcggcggagatacaccggctggggcaggctgagccggaagctgatcaacggcatccgggacaagcagtccggcaagacaatcct
ggatttcctgaagtccgacggcttcgccaacagaaacttcatgcagctgatccacgacgacagcctgacctttaaagaggacatccagaaagcccaggtgtccggccagggcgatagcctgcac
gagcacattgccaatctggccggcagccccgccattaagaagggcatcctgcagacagtgaaggtggtggacgagctcgtgaaagtgatgggccggcacaagcccgagaacatcgtgatcga
aatggccagagagaaccagaccacccagaagggacagaagaacagccgcgagagaatgaagcggatcgaagagggcatcaaagagctgggcagccagatcctgaaagaacaccccgtg
gaaaacacccagctgcagaacgagaagctgtacctgtactacctgcagaatgggcgggatatgtacgtggaccaggaactggacatcaaccggctgtccgactacgatgtggacgctatcgtgc
ctcagagctttctgaaggacgactccatcgacaacaaggtgctgaccagaagcgacaagaaccggggcaagagcgacaacgtgccctccgaagaggtcgtgaagaagatgaagaactactg
gcggcagctgctgaacgccaagctgattacccagagaaagttcgacaatctgaccaaggccgagagaggcggcctgagcgaactggataaggccggcttcatcaagagacagctggtggaa
acccggcagatcacaaagcacgtggcacagatcctggactcccggatgaacactaagtacgacgagaatgacaagctgatccgggaagtgaaagtgatcaccctgaagtccaagctggtgtcc
gatttccggaaggatttccagttttacaaagtgcgcgagatcaacaactaccaccacgcccacgacgcctacctgaacgccgtcgtgggaaccgccctgatcaaaaagtaccctaagctggaaagc
gagttcgtgtacggcgactacaaggtgtacgacgtgcggaagatgatcgccaagagcgagcaggaaatcggcaaggctaccgccaagtacttcttctacagcaacatcatgaactttttcaagac
cgagattaccctggccaacggcgagatccggaagcggcctctgatcgagacaaacggcgaaaccggggagatcgtgtgggataagggccgggattttgccaccgtgcggaaagtgctgagc
atgccccaagtgaatatcgtgaaaaagaccgaggtgcagacaggcggcttcagcaaagagtctatcctgcccaagaggaacagcgataagctgatcgccagaaagaaggactgggaccctaa
gaagtacggcggcttcgacagccccaccgtggcctattctgtgctggtggtggccaaagtggaaaagggcaagtccaagaaactgaagagtgtgaaagagctgctggggatcaccatcatgga
aagaagcagcttcgagaagaatcccatcgactttctggaagccaagggctacaaagaagtgaaaaaggacctgatcatcaagctgcctaagtactccctgttcgagctggaaaacggccggaag
agaatgctggcctctgccggcgaactgcagaagggaaacgaactggccctgccctccaaatatgtgaacttcctgtacctggccagccactatgagaagctgaagggctcccccgaggataatg
agcagaaacagctgtttgtggaacagcacaagcactacctggacgagatcatcgagcagatcagcgagttctccaagagagtgatcctggccgacgctaatctggacaaagtgctgtccgcctac
aacaagcaccgggataagcccatcagagagcaggccgagaatatcatccacctgtttaccctgaccaatctgggagcccctgccgccttcaagtactttgacaccaccatcgaccggaagaggta
caccagcaccaaagaggtgctggacgccaccctgatccaccagagcatcaccggcctgtacgagacacggatcgacctgtctcagctgggaggtgactccggcggaagctctggtggcagcaa
gcggaccgccgacggctctgaattcgagagccctaagaagaaaagaaaggtgagcggaggctctagcggcggaagcaccctgaacattgaagacgagtatagactgcatgaaacaagcaa
ggaacccgacgtgtccctgggctccacctggctgtccgactttccccaggcctgggccgagacaggaggaatgggcctggccgtgcggcaggcacccctgatcatccctctgaaggccacctct
acacccgtgagcatcaagcagtaccctatgtctcaggaggccagactgggcatcaagcctcacatccagaggctgctggaccagggcatcctggtgccatgccagagcccctggaacacaccac
tgctgcccgtgaagaagccaggcaccaatgactatagacccgtgcaggatctgagagaggtgaacaagagggtggaggatatccaccccaccgtgcccaacccttacaatctgctgtccggcct
gcccccttctcaccagtggtatacagtgctggacctgaaggatgccttcttttgtctgagactgcaccctaccagccagccactgttcgcctttgagtggagggaccctgagatgggcatctctggcca
gctgacctggacacgcctgcctcagggcttcaagaatagcccaacactgtttaacgaggccctgcaccgcgacctggcagatttccggatccagcacccagatctgatcctgctgcagtacgtgga
cgatctgctgctggccgccaccagcgagctggattgccagcagggaacacgcgccctgctgcagaccctgggaaacctgggatatagggcatccgccaagaaggcccagatctgtcagaagc
aggtgaagtacctgggctatctgctgaaggagggccagagatggctgacagaggccaggaaggagacagtgatgggccagccaacacccaagaccccaagacagctgagggagttcctgg
gcaaagcaggattttgcaggctgttcatcccaggattcgcagagatggcagcacctctgtacccactgaccaagccgggcaccctgtttaattggggccctgaccagcagaaggcctatcaggag
atcaagcaggccctgctgacagcaccagccctgggcctgccagacctgaccaagcctttcgagctgtttgtggatgagaagcagggctacgccaagggcgtgctgacccagaagctgggacca
tggagacggcccgtggcctatctgtccaagaagctggacccagtggcagcaggatggccaccatgcctgaggatggtggcagcaatcgccgtgctgacaaaggatgccggcaagctgaccat
gggacagccactggtcatcctggcaccacacgcagtggaggccctggtgaagcagcctccagatcgctggctgtctaacgcccggatgacacactaccaggccctgctgctggacaccgatcgc
gtgcagtttggccctgtggtggccctgaatccagccaccctgctgcctctgccagaggagggcctgcagcacaactgtctggacatcctggcagaggcacacggaacaaggccagacctgaccg
atcagcccctgcctgacgccgatcacacatggtataccgatggaagctccctgctgcaggagggccagaggaaggcaggagcagcagtgaccacagagacagaagtgatctgggccaaggc
cctgccagcaggcacatccgcccagcgggccgagctgatcgccctgacccaggccctgaagatggccgagggcaagaagctgaacgtgtacacagactccagatatgccttcgccaccgcaca
catccacggagagatctacaggcgccggggctggctgacctctgagggcaaggagatcaagaacaaggatgagatcctggccctgctgaaggccctgtttctgcccaagcggctgagcatcat
ccactgtcctggacaccagaagggacactccgccgaggcaaggggcaatcggatggccgaccaggccgccagaaaggctgctattactgaaactcccgacacttccactctgctgattgaaaac
tcctcccct

nCas9:

atgaaacggacagccgacggaagcgagttcgagtcaccaaagaagaagcggaaagtcgacaagaagtacagcatcggcctggacatcggcaccaactctgtgggctgggccgtgatcaccg
acgagtacaaggtgcccagcaagaaattcaaggtgctgggcaacaccgaccggcacagcatcaagaagaacctgatcggagccctgctgttcgacagcggcgaaacagccgaggccacccg
gctgaagagaaccgccagaagaagatacaccagacggaagaaccggatctgctatctgcaagagatcttcagcaacgagatggccaaggtggacgacagcttcttccacagactggaagagt
ccttcctggtggaagaggataagaagcacgagcggcaccccatcttcggcaacatcgtggacgaggtggcctaccacgagaagtaccccaccatctaccacctgagaaagaaactggtggaca
gcaccgacaaggccgacctgcggctgatctatctggccctggcccacatgatcaagttccggggccacttcctgatcgagggcgacctgaaccccgacaacagcgacgtggacaagctgttcatc
cagctggtgcagacctacaaccagctgttcgaggaaaaccccatcaacgccagcggcgtggacgccaaggccatcctgtctgccagactgagcaagagcagaaagctggaaaatctgatcgcc
cagctgcccggcgagaagaagaatggcctgttcggaaacctgattgccctgagcctgggcctgacccccaacttcaagagcaacttcgacctggccgaggatgccaaactgcagctgagcaag
gacacctacgacgacgacctggacaacctgctggcccagatcggcgaccagtacgccgacctgtttctggccgccaagaacctgtccgacgccatcctgctgagcgacatcctgagagtgaacac
cgagatcaccaaggcccccctgagcgcctctatgatcaagagatacgacgagcaccaccaggacctgaccctgctgaaagctctcgtgcggcagcagctgcctgagaagtacaaagagattttct

tcgaccagagcaagaacggctacgccggctacattgacggcggagccagccaggaagagttctacaagttcatcaagcccatcctggaaaagatggacggcaccgaggaactgctcgtgaag
ctgaagagagaggacctgctgcggaagcagcggaccttcgacaacggcagcatccccccaccagatccacctgggagagctgcacgccattctgcggcggcaggaagatttttacccattcctga
aggacaaccgggaaaagatcgagaagatcctgaccttccgcatcccctactacgtgggccctctggccaggggaaacagcagattcgcctggatgaccagaaagagcgaggaaaccatcaccc
cctggaacttcgaggaagtggtggacaagggcgcttccgcccagagcttcatcgagcggatgaccaacttcgataagaacctgcccaacgagaaggtgctgcccaagcacagcctgctgtacg
agtacttcaccgtgtataacgagctgaccaaagtgaaatacgtgaccgagggaatgagaaagcccgccttcctgagcggcgagcagaaaaaggccatcgtggacctgctgttcaagaccaacc
ggaaagtgaccgtgaagcagctgaaagaggactacttcaagaaaatcgagtgcttcgactccgtggaaatctccggcgtggaagatcggttcaacgcctccctgggcacataccacgatctgctg
aaaattatcaaggacaaggacttcctggacaatgaggaaaacgaggacattctggaagatatcgtgctgaccctgacactgtttgaggacagagagatgatcgaggaacggctgaaaacctatg
cccacctgttcgacgacaaagtgatgaagcagctgaagcggcggagatacaccggctggggcaggctgagccggaagctgatcaacggcatccgggacaagcagtccggcaagacaatcct
ggatttcctgaagtccgacggcttcgccaacagaaacttcatgcagctgatccacgacgacagcctgacctttaaagaggacatccagaaagcccaggtgtccggccagggcgatagcctgcac
gagcacattgccaatctggccggcagccccgccattaagaagggcatcctgcagacagtgaaggtggtggacgagctcgtgaaagtgatgggccggcacaagcccgagaacatcgtgatcga
aatggccagagagaaccagaccacccagaagggacagaagaacagccgcgagagaatgaagcggatcgaagagggcatcaaagagctgggcagccagatcctgaaagaacaccccgtg
gaaaacacccagctgcagaacgagaagctgtacctgtactacctgcagaatgggcgggatatgtacgtggaccaggaactggacatcaaccggctgtccgactacgatgtggacgctatcgtgc
ctcagagctttctgaaggacgactccatcgacaacaaggtgctgaccagaagcgacaagaaccggggcaagagcgacaacgtgccctccgaagaggtcgtgaagaagatgaagaactactg
gcggcagctgctgaacgccaagctgattacccagagaaagttcgacaatctgaccaaggccgagagaggcggcctgagcgaactggataaggccggcttcatcaagagacagctggtggaa
acccggcagatcacaaagcacgtggcacagatcctggactcccggatgaacactaagtacgacgagaatgacaagctgatccgggaagtgaaagtgatcaccctgaagtccaagctggtgtcc
gatttccggaaggatttccagttttacaaagtgcgcgagatcaacaactaccaccacgcccacgacgcctacctgaacgccgtcgtgggaaccgccctgatcaaaaagtaccctaagctggaaagc
gagttcgtgtacggcgactacaaggtgtacgacgtgcggaagatgatcgccaagagcgagcaggaaatcggcaaggctaccgccaagtacttcttctacagcaacatcatgaactttttcaagac
cgagattaccctggccaacggcgagatccggaagcggcctctgatcgagacaaacggcgaaaccggggagatcgtgtgggataagggccgggattttgccaccgtgcggaaagtgctgagc
atgccccaagtgaatatcgtgaaaaagaccgaggtgcagacaggcggcttcagcaaagagtctatcctgcccaagaggaacagcgataagctgatcgccagaaagaaggactgggaccctaa
gaagtacggcggcttcgacagccccaccgtggcctattctgtgctggtggtggccaaagtggaaaagggcaagtccaagaaactgaagagtgtgaaagagctgctggggatcaccatcatgga
aagaagcagcttcgagaagaatcccatcgactttctggaagccaagggctacaaagaagtgaaaaaggacctgatcatcaagctgcctaagtactccctgttcgagctggaaaacggccggaag
agaatgctggcctctgccggcgaactgcagaagggaaacgaactggccctgccctccaaatatgtgaacttcctgtacctggccagccactatgagaagctgaagggctcccccgaggataatg
agcagaaacagctgtttgtggaacagcacaagcactacctggacgagatcatcgagcagatcagcgagttctccaagagagtgatcctggccgacgctaatctggacaaagtgctgtccgcctac
aacaagcaccgggataagcccatcagagagcaggccgagaatatcatccacctgtttaccctgaccaatctgggagcccctgccgccttcaagtactttgacaccaccatcgaccggaagaggta
caccagcaccaaagaggtgctggacgccaccctgatccaccagagcatcaccggcctgtacgagacacggatcgacctgtctcagctgggaggtgac

RT (for PEmax-SPL):

atgaaacggacagccgacggaagcgagttcgagtcacctaagaagaaaagaaaggtgagcggaggctctagcggcggaagcaccctgaacattgaagacgagtatagactgcatgaaaca
agcaaggaacccgacgtgtccctgggctccacctggctgtccgactttccccaggcctgggccgagacaggaggaatgggcctggccgtgcggcaggcacccctgatcatccctctgaaggcc
acctctacacccgtgagcatcaagcagtaccctatgtctcaggaggccagactgggcatcaagcctcacatccagaggctgctggaccagggcatcctggtgccatgccagagcccctggaacac
accactgctgcccgtgaagaagccaggcaccaatgactatagacccgtgcaggatctgagagaggtgaacaagagggtggaggatatccaccccaccgtgcccaacccttacaatctgctgtcc
ggcctgcccccttctcaccagtggtatacagtgctggacctgaaggatgccttcttttgtctgagactgcaccctaccagccagccactgttcgcctttgagtggagggaccctgagatgggcatctct
ggccagctgacctggacacgcctgcctcagggcttcaagaatagcccaacactgtttaacgaggccctgcaccgcgacctggcagatttccggatccagcacccagatctgatcctgctgcagtac
gtggacgatctgctgctggccgccaccagcgagctggattgccagcagggaacacgcgccctgctgcagaccctgggaaacctgggatatagggcatccgccaagaaggcccagatctgtca
gaagcaggtgaagtacctgggctatctgctgaaggagggccagagatggctgacagaggccaggaaggagacagtgatgggccagccaacacccaagaccccaagacagctgagggagt
tcctgggcaaagcaggattttgcaggctgttcatcccaggattcgcagagatggcagcacctctgtacccactgaccaagccgggcaccctgtttaattggggccctgaccagcagaaggcctatc
aggagatcaagcaggccctgctgacagcaccagccctgggcctgccagacctgaccaagcctttcgagctgtttgtggatgagaagcagggctacgccaagggcgtgctgacccagaagctg
ggaccatggagacggcccgtggcctatctgtccaagaagctggacccagtggcagcaggatggccaccatgcctgaggatggtggcagcaatcgccgtgctgacaaaggatgccggcaagct
gaccatgggacagccactggtcatcctggcaccacacgcagtggaggccctggtgaagcagcctccagatcgctggctgtctaacgcccggatgacacactaccaggccctgctgctggacacc
gatcgcgtgcagtttggccctgtggtggccctgaatccagccaccctgctgcctctgccagaggagggcctgcagcacaactgtctggacatcctggcagaggcacacggaacaaggccagacc
tgaccgatcagcccctgcctgacgccgatcacacatggtataccgatggaagctccctgctgcaggagggccagaggaaggcaggagcagcagtgaccacagagacagaagtgatctgggc
caaggccctgccagcaggcacatccgcccagcgggccgagctgatcgccctgacccaggccctgaagatggccgagggcaagaagctgaacgtgtacacagactccagatatgccttcgccac
cgcacacatccacggagagatctacaggcgccggggctggctgacctctgagggcaaggagatcaagaacaaggatgagatcctggccctgctgaaggccctgtttctgcccaagcggctga
gcatcatccactgtcctggacaccagaagggacactccgccgaggcaaggggcaatcggatggccgaccaggccgccagaaaggctgctattactgaaactcccgacacttccactctgctgatt
gaaaactcctcccct

RTΔRH (for PEmax-SPLΔRH)

atgaaacggacagccgacggaagcgagttcgagtcacctaagaagaaaagaaaggtgagcggaggctctagcggcggaagcaccctgaacattgaagacgagtatagactgcatgaaaca
agcaaggaacccgacgtgtccctgggctccacctggctgtccgactttccccaggcctgggccgagacaggaggaatgggcctggccgtgcggcaggcacccctgatcatccctctgaaggcc
acctctacacccgtgagcatcaagcagtaccctatgtctcaggaggccagactgggcatcaagcctcacatccagaggctgctggaccagggcatcctggtgccatgccagagcccctggaacac
accactgctgcccgtgaagaagccaggcaccaatgactatagacccgtgcaggatctgagagaggtgaacaagagggtggaggatatccaccccaccgtgcccaacccttacaatctgctgtcc
ggcctgcccccttctcaccagtggtatacagtgctggacctgaaggatgccttcttttgtctgagactgcaccctaccagccagccactgttcgcctttgagtggagggaccctgagatgggcatctct
ggccagctgacctggacacgcctgcctcagggcttcaagaatagcccaacactgtttaacgaggccctgcaccgcgacctggcagatttccggatccagcacccagatctgatcctgctgcagtac
gtggacgatctgctgctggccgccaccagcgagctggattgccagcagggaacacgcgccctgctgcagaccctgggaaacctgggatatagggcatccgccaagaaggcccagatctgtca
gaagcaggtgaagtacctgggctatctgctgaaggagggccagagatggctgacagaggccaggaaggagacagtgatgggccagccaacacccaagaccccaagacagctgagggagt
tcctgggcaaagcaggattttgcaggctgttcatcccaggattcgcagagatggcagcacctctgtacccactgaccaagccgggcaccctgtttaattggggccctgaccagcagaaggcctatc
aggagatcaagcaggccctgctgacagcaccagccctgggcctgccagacctgaccaagcctttcgagctgtttgtggatgagaagcagggctacgccaagggcgtgctgacccagaagctg
ggaccatggagacggcccgtggcctatctgtccaagaagctggacccagtggcagcaggatggccaccatgcctgaggatggtggcagcaatcgccgtgctgacaaaggatgccggcaagct
gaccatgggacagccactggtcatcctggcaccacacgcagtggaggccctggtgaagcagcctccagatcgctggctgtctaacgcccggatgacacactaccaggccctgctgctggacacc
gatcgcgtgcagtttggccctgtggtggccctgaatccagccaccctgctgcctctgccagaggagggcctgcagcacaactgtctt

nCas9-GCN4:

atgaaacggacagccgacggaagcgagttcgagtcaccaaagaagaagcggaaagtcgacaagaagtacagcatcggcctggacatcggcaccaactctgtgggctgggccgtgatcaccg
acgagtacaaggtgcccagcaagaaattcaaggtgctgggcaacaccgaccggcacagcatcaagaagaacctgatcggagccctgctgttcgacagcggcgaaacagccgaggccacccg
gctgaagagaaccgccagaagaagatacaccagacggaagaaccggatctgctatctgcaagagatcttcagcaacgagatggccaaggtggacgacagcttcttccacagactggaagagt
ccttcctggtggaagaggataagaagcacgagcggcaccccatcttcggcaacatcgtggacgaggtggcctaccacgagaagtaccccaccatctaccacctgagaaagaaactggtggaca
gcaccgacaaggccgacctgcggctgatctatctggccctggcccacatgatcaagttccggggccacttcctgatcgagggcgacctgaaccccgacaacagcgacgtggacaagctgttcatc
cagctggtgcagacctacaaccagctgttcgaggaaaaccccatcaacgccagcggcgtggacgccaaggccatcctgtctgccagactgagcaagagcagaaagctggaaaatctgatcgcc
cagctgcccggcgagaagaagaatggcctgttcggaaacctgattgccctgagcctgggcctgacccccaacttcaagagcaacttcgacctggccgaggatgccaaactgcagctgagcaag
gacacctacgacgacgacctggacaacctgctggcccagatcggcgaccagtacgccgacctgtttctggccgccaagaacctgtccgacgccatcctgctgagcgacatcctgagagtgaacac
cgagatcaccaaggcccccctgagcgcctctatgatcaagagatacgacgagcaccaccaggacctgaccctgctgaaagctctcgtgcggcagcagctgcctgagaagtacaaagagattttct
tcgaccagagcaagaacggctacgccggctacattgacggcggagccagccaggaagagttctacaagttcatcaagcccatcctggaaaagatggacggcaccgaggaactgctcgtgaag
ctgaagagagaggacctgctgcggaagcagcggaccttcgacaacggcagcatcccccaccagatccacctgggagagctgcacgccattctgcggcggcaggaagatttttacccattcctga
aggacaaccgggaaaagatcgagaagatcctgaccttccgcatcccctactacgtgggccctctggccaggggaaacagcagattcgcctggatgaccagaaagagcgaggaaaccatcaccc
cctggaacttcgaggaagtggtggacaagggcgcttccgcccagagcttcatcgagcggatgaccaacttcgataagaacctgcccaacgagaaggtgctgcccaagcacagcctgctgtacg
agtacttcaccgtgtataacgagctgaccaaagtgaaatacgtgaccgagggaatgagaaagcccgccttcctgagcggcgagcagaaaaaggccatcgtggacctgctgttcaagaccaacc
ggaaagtgaccgtgaagcagctgaaagaggactacttcaagaaaatcgagtgcttcgactccgtggaaatctccggcgtggaagatcggttcaacgcctccctgggcacataccacgatctgctg
aaaattatcaaggacaaggacttcctggacaatgaggaaaacgaggacattctggaagatatcgtgctgaccctgacactgtttgaggacagagagatgatcgaggaacggctgaaaacctatg
cccacctgttcgacgacaaagtgatgaagcagctgaagcggcggagatacaccggctggggcaggctgagccggaagctgatcaacggcatccgggacaagcagtccggcaagacaatcct
ggatttcctgaagtccgacggcttcgccaacagaaacttcatgcagctgatccacgacgacagcctgacctttaaagaggacatccagaaagcccaggtgtccggccagggcgatagcctgcac
gagcacattgccaatctggccggcagccccgccattaagaagggcatcctgcagacagtgaaggtggtggacgagctcgtgaaagtgatgggccggcacaagcccgagaacatcgtgatcga
aatggccagagagaaccagaccacccagaagggacagaagaacagccgcgagagaatgaagcggatcgaagagggcatcaaagagctgggcagccagatcctgaaagaacaccccgtg
gaaaacacccagctgcagaacgagaagctgtacctgtactacctgcagaatgggcgggatatgtacgtggaccaggaactggacatcaaccggctgtccgactacgatgtggacgctatcgtgc
ctcagagctttctgaaggacgactccatcgacaacaaggtgctgaccagaagcgacaagaaccggggcaagagcgacaacgtgccctccgaagaggtcgtgaagaagatgaagaactactg
gcggcagctgctgaacgccaagctgattacccagagaaagttcgacaatctgaccaaggccgagagaggcggcctgagcgaactggataaggccggcttcatcaagagacagctggtggaa
acccggcagatcacaaagcacgtggcacagatcctggactcccggatgaacactaagtacgacgagaatgacaagctgatccgggaagtgaaagtgatcaccctgaagtccaagctggtgtcc
gatttccggaaggatttccagttttacaaagtgcgcgagatcaacaactaccaccacgcccacgacgcctacctgaacgccgtcgtgggaaccgccctgatcaaaaagtaccctaagctggaaagc
gagttcgtgtacggcgactacaaggtgtacgacgtgcggaagatgatcgccaagagcgagcaggaaatcggcaaggctaccgccaagtacttcttctacagcaacatcatgaactttttcaagac
cgagattaccctggccaacggcgagatccggaagcggcctctgatcgagacaaacggcgaaaccggggagatcgtgtgggataagggccgggattttgccaccgtgcggaaagtgctgagc
atgccccaagtgaatatcgtgaaaaagaccgaggtgcagacaggcggcttcagcaaagagtctatcctgcccaagaggaacagcgataagctgatcgccagaaagaaggactgggaccctaa
gaagtacggcggcttcgacagccccaccgtggcctattctgtgctggtggtggccaaagtggaaaagggcaagtccaagaaactgaagagtgtgaaagagctgctggggatcaccatcatgga
aagaagcagcttcgagaagaatcccatcgactttctggaagccaagggctacaaagaagtgaaaaaggacctgatcatcaagctgcctaagtactccctgttcgagctggaaaacggccggaag
agaatgctggcctctgccggcgaactgcagaagggaaacgaactggccctgccctccaaatatgtgaacttcctgtacctggccagccactatgagaagctgaagggctcccccgaggataatg
agcagaaacagctgtttgtggaacagcacaagcactacctggacgagatcatcgagcagatcagcgagttctccaagagagtgatcctggccgacgctaatctggacaaagtgctgtccgcctac
aacaagcaccgggataagcccatcagagagcaggccgagaatatcatccacctgtttaccctgaccaatctgggagcccctgccgccttcaagtactttgacaccaccatcgaccggaagaggta
caccagcaccaaagaggtgctggacgccaccctgatccaccagagcatcaccggcctgtacgagacacggatcgacctgtctcagctgggaggtgactctggaggatctagcggaggatccgg
cggttctggttccggctcaggtggaagtggatctggcggctcaggttccggcgaagaattgctttccaagaactaccatttggaaaatgaagtcgctcgtttgaagaaa

scFv-RT (for PEmax-SPL-tag):

atgaaacggacagccgacggaagcgagttcgagtcacctaagaagaaaagaaaggtgtctagaatgggtcccgacatcgtgatgacccagagccccagcagcctgagcgccagcgtgggcg
accgcgtgaccatcacctgccgcagcagcaccggcgccgtgaccaccagcaactacgccagctgggtgcaggagaagcccggcaagctgttcaagggcctgatcggcggcaccaacaaccg
cgcccccggcgtgcccagccgcttcagcggcagcctgatcggcgacaaggccaccctgaccatcagcagcctgcagcccgaggacttcgccacctacttctgcgccctgtggtacagcaaccact
gggtgttcggccagggcaccaaggtggagctgaagcgcggcggcggcggcagcagcggcggcggcagcgaggtgaagctgctggagagcggcggcggcctggtgcagcccggcggc
agcctgaagctgagctgcgccgtgagcggcttcagcctgaccgactacggcgtgaactgggtgcgccaggcccccggccgcggcctggagtggatcggcgtgatctggggcgacggcatca
ccgactacaacagcgccctgaaggaccgcttcatcatcagcaaggacaacggcaagaacaccgtgtacctgcagatgagcaaggtgcgcagcgacgacaccgccctgtactactgcgtgaccg
gcctgttcgactactggggccagggcaccctggtgaccgtgagcagcggatcctctggcagcgagacaccaggaacaagcgagtcagcaacaccagagagcagtggcggcagcagcggcg
gcagcagcagcaccctgaacattgaagacgagtatagactgcatgaaacaagcaaggaacccgacgtgtccctgggctccacctggctgtccgactttccccaggcctgggccgagacaggag
gaatgggcctggccgtgcggcaggcacccctgatcatccctctgaaggccacctctacacccgtgagcatcaagcagtaccctatgtctcaggaggccagactgggcatcaagcctcacatccag
aggctgctggaccagggcatcctggtgccatgccagagcccctggaacacaccactgctgcccgtgaagaagccaggcaccaatgactatagacccgtgcaggatctgagagaggtgaacaa
gagggtggaggatatccaccccaccgtgcccaacccttacaatctgctgtccggcctgccccctctcaccagtggtatacagtgctggacctgaaggatgccttcttttgtctgagactgcaccctac
cagccagccactgttcgcctttgagtggagggaccctgagatgggcatctctggccagctgacctggacacgcctgcctcagggcttcaagaatagcccaacactgtttaacgaggccctgcaccg
cgacctggcagatttccggatccagcacccagatctgatcctgctgcagtacgtggacgatctgctgctggccgccaccagcgagctggattgccagcagggaacacgcgccctgctgcagaccc
tgggaaacctgggatatagggcatccgccaagaaggcccagatctgtcagaagcaggtgaagtacctgggctatctgctgaaggagggccagagatggctgacagaggccaggaaggaga
cagtgatgggccagccaacacccaagaccccaagacagctgagggagttcctgggcaaagcaggattttgcaggctgttcatcccaggattcgcagagatggcagcacctctgtacccactgac
caagccgggcaccctgtttaattggggccctgaccagcagaaggcctatcaggagatcaagcaggccctgctgacagcaccagccctgggcctgccagacctgaccaagcctttcgagctgtttg
tggatgagaagcagggctacgccaagggcgtgctgacccagaagctgggaccatggagacggcccgtggcctatctgtccaagaagctggacccagtggcagcaggatggccaccatgcct
gaggatggtggcagcaatcgccgtgctgacaaaggatgccggcaagctgaccatgggacagccactggtcatcctggcaccacacgcagtggaggccctggtgaagcagcctccagatcgct
ggctgtctaacgcccggatgacacactaccaggccctgctgctggacaccgatcgcgtgcagtttggccctgtggtggccctgaatccagccaccctgctgcctctgccagaggagggcctgcag
cacaactgtctggacatcctggcagaggcacacggaacaaggccagacctgaccgatcagcccctgcctgacgccgatcacacatggtataccgatggaagctccctgctgcaggagggccag
aggaaggcaggagcagcagtgaccacagagacagaagtgatctgggccaaggccctgccagcaggcacatccgcccagcgggccgagctgatcgccctgacccaggccctgaagatggcc
gagggcaagaagctgaacgtgtacacagactccagatatgccttcgccaccgcacacatccacggagagatctacaggcgccggggctggctgacctctgagggcaaggagatcaagaacaa
ggatgagatcctggccctgctgaaggccctgtttctgcccaagcggctgagcatcatccactgtcctggacaccagaaggacactccgccgaggcaaggggcaatcggatggccgaccaggc
cgccagaaaggctgctattactgaaactcccgacacttccactctgctgattgaaaactcctcccct

scFv-RTΔRH (for PEmax-SPLΔRH-tag):

atgaaacggacagccgacggaagcgagttcgagtcacctaagaagaaaagaaaggtgtctagaatgggtcccgacatcgtgatgacccagagccccagcagcctgagcgccagcgtgggcg
accgcgtgaccatcacctgccgcagcagcaccggcgccgtgaccaccagcaactacgccagctgggtgcaggagaagcccggcaagctgttcaagggcctgatcggcggcaccaacaaccg
cgcccccggcgtgcccagccgcttcagcggcagcctgatcggcgacaaggccaccctgaccatcagcagcctgcagcccgaggacttcgccacctacttctgcgccctgtggtacagcaaccact
gggtgttcggccagggcaccaaggtggagctgaagcgcggcggcggcggcagcagcggcggcggcagcgaggtgaagctgctggagagcggcggcggcctggtgcagcccggcggc
agcctgaagctgagctgcgccgtgagcggcttcagcctgaccgactacggcgtgaactgggtgcgccaggcccccggccgcggcctggagtggatcggcgtgatctggggcgacggcatca
ccgactacaacagcgccctgaaggaccgcttcatcatcagcaaggacaacggcaagaacaccgtgtacctgcagatgagcaaggtgcgcagcgacgacaccgccctgtactactgcgtgaccg
gcctgttcgactactggggccagggcaccctggtgaccgtgagcagcggatcctctggcagcgagacaccaggaacaagcgagtcagcaacaccagagagcagtggcggcagcagcggcg
gcagcagcagcaccctgaacattgaagacgagtatagactgcatgaaacaagcaaggaacccgacgtgtccctgggctccacctggctgtccgactttccccaggcctgggccgagacaggag
gaatgggcctggccgtgcggcaggcacccctgatcatccctctgaaggccacctctacacccgtgagcatcaagcagtaccctatgtctcaggaggccagactgggcatcaagcctcacatccag
aggctgctggaccagggcatcctggtgccatgccagagcccctggaacacaccactgctgcccgtgaagaagccaggcaccaatgactatagacccgtgcaggatctgagagaggtgaacaa
gagggtggaggatatccaccccaccgtgcccaacccttacaatctgctgtccggcctgccccccttctcaccagtggtatacagtgctggacctgaaggatgccttcttttgtctgagactgcaccctac
cagccagccactgttcgcctttgagtggagggaccctgagatgggcatctctggccagctgacctggacacgcctgcctcagggcttcaagaatagcccaacactgtttaacgaggccctgcaccg
cgacctggcagatttccggatccagcacccagatctgatcctgctgcagtacgtggacgatctgctgctggccgccaccagcgagctggattgccagcagggaacacgcgccctgctgcagaccc
tgggaaacctgggatatagggcatccgccaagaaggcccagatctgtcagaagcaggtgaagtacctgggctatctgctgaaggagggccagagatggctgacagaggccaggaaggaga
cagtgatgggccagccaacacccaagaccccaagacagctgagggagttcctgggcaaagcaggattttgcaggctgttcatcccaggattcgcagagatggcagcacctctgtacccactgac
caagccgggcaccctgtttaattggggccctgaccagcagaaggcctatcaggagatcaagcaggccctgctgacagcaccagccctgggcctgccagacctgaccaagcctttcgagctgtttg
tggatgagaagcagggctacgccaagggcgtgctgacccagaagctgggaccatggagacggcccgtggcctatctgtccaagaagctggacccagtggcagcaggatggccaccatgcct
gaggatggtggcagcaatcgccgtgctgacaaaggatgccggcaagctgaccatgggacagccactggtcatcctggcaccacacgcagtggaggccctggtgaagcagcctccagatcgct
ggctgtctaacgcccggatgacacactaccaggccctgctgctggacaccgatcgcgtgcagtttggccctgtggtggccctgaatccagccaccctgctgcctctgccagaggagggcctgcag
cacaactgtctt

uPEn (Cas9-RT-P2A-i53):

atgaaacggacagccgacggaagcgagttcgagtcaccaaagaagaagcggaaagtcgacaagaagtacagcatcggcctggacatcggcaccaactctgtgggctgggccgtgatcaccg
acgagtacaaggtgcccagcaagaaattcaaggtgctgggcaacaccgaccggcacagcatcaagaagaacctgatcggagccctgctgttcgacagcggcgaaacagccgaggccacccg
gctgaagagaaccgccagaagaagatacaccagacggaagaaccggatctgctatctgcaagagatcttcagcaacgagatggccaaggtggacgacagcttcttccacagactggaagagt
ccttcctggtggaagaggataagaagcacgagcggcaccccatcttcggcaacatcgtggacgaggtggcctaccacgagaagtaccccaccatctaccacctgagaaagaaactggtggaca
gcaccgacaaggccgacctgcggctgatctatctggccctggcccacatgatcaagttccggggccacttcctgatcgagggcgacctgaaccccgacaacagcgacgtggacaagctgttcatc
cagctggtgcagacctacaaccagctgttcgaggaaaaccccatcaacgccagcggcgtggacgccaaggccatcctgtctgccagactgagcaagagcagaaagctggaaaatctgatcgcc
cagctgcccggcgagaagaagaatggcctgttcggaaacctgattgccctgagcctgggcctgacccccaacttcaagagcaacttcgacctggccgaggatgccaaactgcagctgagcaag
gacacctacgacgacgacctggacaacctgctggcccagatcggcgaccagtacgccgacctgtttctggccgccaagaacctgtccgacgccatcctgctgagcgacatcctgagagtgaacac
cgagatcaccaaggcccccctgagcgcctctatgatcaagagatacgacgagcaccaccaggacctgaccctgctgaaagctctcgtgcggcagcagctgcctgagaagtacaaagagattttct
tcgaccagagcaagaacggctacgccggctacattgacggcggagccagccaggaagagttctacaagttcatcaagcccatcctggaaaagatggacggcaccgaggaactgctcgtgaag
ctgaagagagaggacctgctgcggaagcagcggaccttcgacaacggcagcatcccccaccagatccacctgggagagctgcacgccattctgcggcggcaggaagatttttacccattcctga
aggacaaccgggaaaagatcgagaagatcctgaccttccgcatcccctactacgtgggccctctggccaggggaaacagcagattcgcctggatgaccagaaagagcgaggaaaccatcaccc
cctggaacttcgaggaagtggtggacaagggcgcttccgcccagagcttcatcgagcggatgaccaacttcgataagaacctgcccaacgagaaggtgctgcccaagcacagcctgctgtacg
agtacttcaccgtgtataacgagctgaccaaagtgaaatacgtgaccgagggaatgagaaagcccgccttcctgagcggcgagcagaaaaaggccatcgtggacctgctgttcaagaccaacc
ggaaagtgaccgtgaagcagctgaaagaggactacttcaagaaaatcgagtgcttcgactccgtggaaatctccggcgtggaagatcggttcaacgcctccctgggcacataccacgatctgctg
aaaattatcaaggacaaggacttcctggacaatgaggaaaacgaggacattctggaagatatcgtgctgaccctgacactgtttgaggacagagagatgatcgaggaacggctgaaaacctatg
cccacctgttcgacgacaaagtgatgaagcagctgaagcggcggagatacaccggctggggcaggctgagccggaagctgatcaacggcatccgggacaagcagtccggcaagacaatcct
ggatttcctgaagtccgacggcttcgccaacagaaacttcatgcagctgatccacgacgacagcctgacctttaaagaggacatccagaaagcccaggtgtccggccagggcgatagcctgcac
gagcacattgccaatctggccggcagccccgccattaagaagggcatcctgcagacagtgaaggtggtggacgagctcgtgaaagtgatgggccggcacaagcccgagaacatcgtgatcga
aatggccagagagaaccagaccacccagaagggacagaagaacagccgcgagagaatgaagcggatcgaagagggcatcaaagagctgggcagccagatcctgaaagaacaccccgtg
gaaaacacccagctgcagaacgagaagctgtacctgtactacctgcagaatgggcgggatatgtacgtggaccaggaactggacatcaaccggctgtccgactacgatgtggacCAtatcgtg
cctcagagctttctgaaggacgactccatcgacaacaaggtgctgaccagaagcgacaagaaccggggcaagagcgacaacgtgccctccgaagaggtcgtgaagaagatgaagaactactg
gcggcagctgctgaacgccaagctgattacccagagaaagttcgacaatctgaccaaggccgagagaggcggcctgagcgaactggataaggccggcttcatcaagagacagctggtggaa
acccggcagatcacaaagcacgtggcacagatcctggactcccggatgaacactaagtacgacgagaatgacaagctgatccgggaagtgaaagtgatcaccctgaagtccaagctggtgtcc
gatttccggaaggatttccagttttacaaagtgcgcgagatcaacaactaccaccacgcccacgacgcctacctgaacgccgtcgtgggaaccgccctgatcaaaaagtaccctaagctggaaagc
gagttcgtgtacggcgactacaaggtgtacgacgtgcggaagatgatcgccaagagcgagcaggaaatcggcaaggctaccgccaagtacttcttctacagcaacatcatgaactttttcaagac
cgagattaccctggccaacggcgagatccggaagcggcctctgatcgagacaaacggcgaaaccggggagatcgtgtgggataagggccgggattttgccaccgtgcggaaagtgctgagc
atgccccaagtgaatatcgtgaaaaagaccgaggtgcagacaggcggcttcagcaaagagtctatcctgcccaagaggaacagcgataagctgatcgccagaaagaaggactgggaccctaa
gaagtacggcggcttcgacagccccaccgtggcctattctgtgctggtggtggccaaagtggaaaagggcaagtccaagaaactgaagagtgtgaaagagctgctggggatcaccatcatgga
aagaagcagcttcgagaagaatcccatcgactttctggaagccaagggctacaaagaagtgaaaaaggacctgatcatcaagctgcctaagtactccctgttcgagctggaaaacggccggaag
agaatgctggcctctgccggcgaactgcagaagggaaacgaactggccctgccctccaaatatgtgaacttcctgtacctggccagccactatgagaagctgaagggctcccccgaggataatg
agcagaaacagctgtttgtggaacagcacaagcactacctggacgagatcatcgagcagatcagcgagttctccaagagagtgatcctggccgacgctaatctggacaaagtgctgtccgcctac
aacaagcaccgggataagcccatcagagagcaggccgagaatatcatccacctgtttaccctgaccaatctgggagcccctgccgccttcaagtactttgacaccaccatcgaccggaagaggta
caccagcaccaaagaggtgctggacgccaccctgatccaccagagcatcaccggcctgtacgagacacggatcgacctgtctcagctgggaggtgactccggcggaagctctggtggcagcaa
gcggaccgccgacggctctgaattcgagagccctaagaagaaaagaaaggtgagcggaggctctagcggcggaagcaccctgaacattgaagacgagtatagactgcatgaaacaagcaa
ggaacccgacgtgtccctgggctccacctggctgtccgactttccccaggcctgggccgagacaggaggaatgggcctggccgtgcggcaggcacccctgatcatccctctgaaggccacctct
acacccgtgagcatcaagcagtaccctatgtctcaggaggccagactgggcatcaagcctcacatccagaggctgctggaccagggcatcctggtgccatgccagagcccctggaacacaccac
tgctgcccgtgaagaagccaggcaccaatgactatagacccgtgcaggatctgagagaggtgaacaagagggtggaggatatccaccccaccgtgcccaacccttacaatctgctgtccggcct
gccccccttctcaccagtggtatacagtgctggacctgaaggatgccttcttttgtctgagactgcaccctaccagccagccactgttcgcctttgagtggagggaccctgagatgggcatctctggcca
gctgacctggacacgcctgcctcagggcttcaagaatagcccaacactgtttaacgaggccctgcaccgcgacctggcagatttccggatccagcacccagatctgatcctgctgcagtacgtgga
cgatctgctgctggccgccaccagcgagctggattgccagcagggaacacgcgccctgctgcagaccctgggaaacctgggatatagggcatccgccaagaaggcccagatctgtcagaagc
aggtgaagtacctgggctatctgctgaaggagggccagagatggctgacagaggccaggaaggagacagtgatgggccagccaacacccaagaccccaagacagctgagggagttcctgg
gcaaagcaggattttgcaggctgttcatcccaggattcgcagagatggcagcacctctgtacccactgaccaagccgggcaccctgtttaattggggccctgaccagcagaaggcctatcaggag

atcaagcaggccctgctgacagcaccagccctgggcctgccagacctgaccaagcctttcgagctgtttgtggatgagaagcagggctacgccaagggcgtgctgacccagaagctgggacca
tggagacggcccgtggcctatctgtccaagaagctggacccagtggcagcaggatggccaccatgcctgaggatggtggcagcaatcgccgtgctgacaaaggatgccggcaagctgaccat
gggacagccactggtcatcctggcaccacacgcagtggaggccctggtgaagcagcctccagatcgctggctgtctaacgcccggatgacacactaccaggccctgctgctggacaccgatcgc
gtgcagtttggccctgtggtggccctgaatccagccaccctgctgcctctgccagaggagggcctgcagcacaactgtctggacatcctggcagaggcacacggaacaaggccagacctgaccg
atcagcccctgcctgacgccgatcacacatggtataccgatggaagctccctgctgcaggagggccagaggaaggcaggagcagcagtgaccacagagacagaagtgatctgggccaaggc
cctgccagcaggcacatccgcccagcgggccgagctgatcgccctgacccaggccctgaagatggccgagggcaagaagctgaacgtgtacacagactccagatatgccttcgccaccgcaca
catccacggagagatctacaggcgccggggctggctgacctctgagggcaaggagatcaagaacaaggatgagatcctggccctgctgaaggccctgtttctgcccaagcggctgagcatcat
ccactgtcctggacaccagaagggacactccgccgaggcaaggggcaatcggatggccgaccaggccgccagaaaggctgctattactgaaactcccgacacttccactctgctgattgaaaac
tcctcccct tctggcggctcaaaaagaaccgccgacggcagcgaattcgagtctcccaagaagaagaggaaagtcggctctggccctgccgctaagagagtgaagctggacGGATCCGGC
GCAACAAACTTCTCTCTGCTGAAACAAGCCGGAGATGTCGAAGAGAATCCTGGACCGATGCATATGaaacggacagccgacggaagcgagttcgagtcaccaa
agaagaagcggaaagtcgccgccagtttaaacggcgcgccattaattaaggatccaatgttgattttcgtgaaaacccttaccgggaaaaccatcaccctcgaggttgaaccctcggatacgatag
aaaatgtaaaggccaagatccaggataaggaaggaattcctcctgatcagcagagactggcctttgctggcaaatcgctggaagatggacgtactttgtctgactacaatattctaaaggactctaa
acttcatcctctgttgagacttcgttaa

Cas9-GCN4:

atgaaacggacagccgacggaagcgagttcgagtcaccaaagaagaagcggaaagtcgacaagaagtacagcatcggcctggacatcggcaccaactctgtgggctgggccgtgatcaccg
acgagtacaaggtgcccagcaagaaattcaaggtgctgggcaacaccgaccggcacagcatcaagaagaacctgatcggagccctgctgttcgacagcggcgaaacagccgaggccacccg
gctgaagagaaccgccagaagaagatacaccagacggaagaaccggatctgctatctgcaagagatcttcagcaacgagatggccaaggtggacgacagcttcttccacagactggaagagt
ccttcctggtggaagaggataagaagcacgagcggcaccccatcttcggcaacatcgtggacgaggtggcctaccacgagaagtaccccaccatctaccacctgagaaagaaactggtggaca
gcaccgacaaggccgacctgcggctgatctatctggccctggcccacatgatcaagttccggggccacttcctgatcgagggcgacctgaaccccgacaacagcgacgtggacaagctgttcatc
cagctggtgcagacctacaaccagctgttcgaggaaaaccccatcaacgccagcggcgtggacgccaaggccatcctgtctgccagactgagcaagagcagaaagctggaaaatctgatcgcc
cagctgcccggcgagaagaagaatggcctgttcggaaacctgattgccctgagcctgggcctgacccccaacttcaagagcaacttcgacctggccgaggatgccaaactgcagctgagcaag
gacacctacgacgacgacctggacaacctgctggcccagatcggcgaccagtacgccgacctgtttctggccgccaagaacctgtccgacgccatcctgctgagcgacatcctgagagtgaacac
cgagatcaccaaggcccccctgagcgcctctatgatcaagagatacgacgagcaccaccaggacctgaccctgctgaaagctctcgtgcggcagcagctgcctgagaagtacaaagagattttct
tcgaccagagcaagaacggctacgccggctacattgacggcggagccagccaggaagagttctacaagttcatcaagcccatcctggaaaagatggacggcaccgaggaactgctcgtgaag
ctgaagagagaggacctgctgcggaagcagcggaccttcgacaacggcagcatccccccaccagatccacctgggagagctgcacgccattctgcggcggcaggaagatttttacccattcctga
aggacaaccgggaaaagatcgagaagatcctgaccttccgcatcccctactacgtgggccctctggccaggggaaacagcagattcgcctggatgaccagaaagagcgaggaaaccatcaccc
cctggaacttcgaggaagtggtggacaagggcgcttccgcccagagcttcatcgagcggatgaccaacttcgataagaacctgcccaacgagaaggtgctgcccaagcacagcctgctgtacg
agtacttcaccgtgtataacgagctgaccaaagtgaaatacgtgaccgagggaatgagaaagcccgccttcctgagcggcgagcagaaaaaggccatcgtggacctgctgttcaagaccaacc
ggaaagtgaccgtgaagcagctgaaagaggactacttcaagaaaatcgagtgcttcgactccgtggaaatctccggcgtggaagatcggttcaacgcctccctgggcacataccacgatctgctg
aaaattatcaaggacaaggacttcctggacaatgaggaaaacgaggacattctggaagatatcgtgctgaccctgacactgtttgaggacagagagatgatcgaggaacggctgaaaacctatg
cccacctgttcgacgacaaagtgatgaagcagctgaagcggcggagatacaccggctggggcaggctgagccggaagctgatcaacggcatccgggacaagcagtccggcaagacaatcct
ggatttcctgaagtccgacggcttcgccaacagaaacttcatgcagctgatccacgacgacagcctgacctttaaagaggacatccagaaagcccaggtgtccggccagggcgatagcctgcac
gagcacattgccaatctggccggcagccccgccattaagaagggcatcctgcagacagtgaaggtggtggacgagctcgtgaaagtgatgggccggcacaagcccgagaacatcgtgatcga
aatggccagagagaaccagaccacccagaagggacagaagaacagccgcgagagaatgaagcggatcgaagagggcatcaaagagctgggcagccagatcctgaaagaacaccccgtg
gaaaacacccagctgcagaacgagaagctgtacctgtactacctgcagaatgggcgggatatgtacgtggaccaggaactggacatcaaccggctgtccgactacgatgtggaccatatcgtgc
ctcagagctttctgaaggacgactccatcgacaacaaggtgctgaccagaagcgacaagaaccggggcaagagcgacaacgtgccctccgaagaggtcgtgaagaagatgaagaactactg
gcggcagctgctgaacgccaagctgattacccagagaaagttcgacaatctgaccaaggccgagagaggcggcctgagcgaactggataaggccggcttcatcaagagacagctggtggaa
acccggcagatcacaaagcacgtggcacagatcctggactcccggatgaacactaagtacgacgagaatgacaagctgatccgggaagtgaaagtgatcaccctgaagtccaagctggtgtcc
gatttccggaaggatttccagttttacaaagtgcgcgagatcaacaactaccaccacgcccacgacgcctacctgaacgccgtcgtgggaaccgccctgatcaaaaagtaccctaagctggaaagc
gagttcgtgtacggcgactacaaggtgtacgacgtgcggaagatgatcgccaagagcgagcaggaaatcggcaaggctaccgccaagtacttcttctacagcaacatcatgaactttttcaagac
cgagattaccctggccaacggcgagatccggaagcggcctctgatcgagacaaacggcgaaaccggggagatcgtgtgggataagggccgggattttgccaccgtgcggaaagtgctgagc
atgccccaagtgaatatcgtgaaaaagaccgaggtgcagacaggcggcttcagcaaagagtctatcctgcccaagaggaacagcgataagctgatcgccagaaagaaggactgggaccctaa
gaagtacggcggcttcgacagccccaccgtggcctattctgtgctggtggtggccaaagtggaaaagggcaagtccaagaaactgaagagtgtgaaagagctgctggggatcaccatcatgga
aagaagcagcttcgagaagaatcccatcgactttctggaagccaagggctacaaagaagtgaaaaaggacctgatcatcaagctgcctaagtactccctgttcgagctggaaaacggccggaag
agaatgctggcctctgccggcgaactgcagaagggaaacgaactggccctgccctccaaatatgtgaacttcctgtacctggccagccactatgagaagctgaagggctcccccgaggataatg
agcagaaacagctgtttgtggaacagcacaagcactacctggacgagatcatcgagcagatcagcgagttctccaagagagtgatcctggccgacgctaatctggacaaagtgctgtccgcctac
aacaagcaccgggataagcccatcagagagcaggccgagaatatcatccacctgtttaccctgaccaatctgggagcccctgccgccttcaagtactttgacaccaccatcgaccggaagaggta
caccagcaccaaagaggtgctggacgccaccctgatccaccagagcatcaccggcctgtacgagacacggatcgacctgtctcagctgggaggtgactctggaggatctagcggaggatccgg
cggttctggttccggctcaggtggaagtggatctggcggctcaggttccggcgaagaattgctttccaagaactaccatttggaaaatgaagtcgctcgtttgaagaaa

scFv-RTΔRH-P2A-i53 (for uPEn-SPLΔRH-tag):

atgaaacggacagccgacggaagcgagttcgagtcacctaagaagaaaagaaaggtgtctagaatgggtcccgacatcgtgatgacccagagccccagcagcctgagcgccagcgtgggcg
accgcgtgaccatcacctgccgcagcagcaccggcgccgtgaccaccagcaactacgccagctgggtgcaggagaagcccggcaagctgttcaagggcctgatcggcggcaccaacaaccg
cgcccccggcgtgcccagccgcttcagcggcagcctgatcggcgacaaggccaccctgaccatcagcagcctgcagcccgaggacttcgccacctacttctgcgccctgtggtacagcaaccact
gggtgttcggccagggcaccaaggtggagctgaagcgcggcggcggcggcagcagcggcggcggcagcgaggtgaagctgctggagagcggcggcggcctggtgcagcccggcggc
agcctgaagctgagctgcgccgtgagcggcttcagcctgaccgactacggcgtgaactgggtgcgccaggcccccggccgcggcctggagtggatcggcgtgatctggggcgacggcatca
ccgactacaacagcgccctgaaggaccgcttcatcatcagcaaggacaacggcaagaacaccgtgtacctgcagatgagcaaggtgcgcagcgacgacaccgccctgtactactgcgtgaccg
gcctgttcgactactggggccagggcaccctggtgaccgtgagcagcggatcctctggcagcgagacaccaggaacaagcgagtcagcaacaccagagagcagtggcggcagcagcggcg
gcagcagcagcaccctgaacattgaagacgagtatagactgcatgaaacaagcaaggaacccgacgtgtccctgggctccacctggctgtccgactttccccaggcctgggccgagacaggag
gaatgggcctggccgtgcggcaggcacccctgatcatccctctgaaggccacctctacacccgtgagcatcaagcagtaccctatgtctcaggaggccagactgggcatcaagcctcacatccag
aggctgctggaccagggcatcctggtgccatgccagagcccctggaacacaccactgctgcccgtgaagaagccaggcaccaatgactatagacccgtgcaggatctgagagaggtgaacaa
gagggtggaggatatccaccccaccgtgcccaacccttacaatctgctgtccggcctgccccccttctcaccagtggtatacagtgctggacctgaaggatgccttcttttgtctgagactgcaccctac
cagccagccactgttcgcctttgagtggagggaccctgagatgggcatctctggccagctgacctggacacgcctgcctcagggcttcaagaatagcccaacactgtttaacgaggccctgcaccg

cgacctggcagatttccggatccagcacccagatctgatcctgctgcagtacgtggacgatctgctgctggccgccaccagcgagctggattgccagcagggaacacgcgccctgctgcagaccc
tgggaaacctgggatatagggcatccgccaagaaggcccagatctgtcagaagcaggtgaagtacctgggctatctgctgaaggagggccagagatggctgacagaggccaggaaggaga
cagtgatgggccagccaacacccaagaccccaagacagctgagggagttcctgggcaaagcaggattttgcaggctgttcatcccaggattcgcagagatggcagcacctctgtacccactgac
caagccgggcaccctgtttaattggggccctgaccagcagaaggcctatcaggagatcaagcaggccctgctgacagcaccagccctgggcctgccagacctgaccaagcctttcgagctgtttg
tggatgagaagcagggctacgccaagggcgtgctgacccagaagctgggaccatggagacggcccgtggcctatctgtccaagaagctggacccagtggcagcaggatggccaccatgcct
gaggatggtggcagcaatcgccgtgctgacaaaggatgccggcaagctgaccatgggacagccactggtcatcctggcaccacacgcagtggaggccctggtgaagcagcctccagatcgct
ggctgtctaacgcccggatgacacactaccaggccctgctgctggacaccgatcgcgtgcagtttggccctgtggtggccctgaatccagccaccctgctgcctctgccagaggagggcctgcag
cacaactgtctttctggcggctcaaaaagaaccgccgacggcagcgaattcgagtctcccaagaagaagaggaaagtcggctctggccctgccgctaagagagtgaagctggacGGATCCG
GCGCAACAAACTTCTCTCTGCTGAAACAAGCCGGAGATGTCGAAGAGAATCCTGGACCGATGCATATGaaacggacagccgacggaagcgagttcgagtcac
caaagaagaagcggaaagtcgccgccagtttaaacggcgcgccattaattaaggatccaatgttgattttcgtgaaaacccttaccgggaaaaccatcaccctcgaggttgaaccctcggatacga
tagaaaatgtaaaggccaagatccaggataaggaaggaattcctcctgatcagcagagactggcctttgctggcaaatcgctggaagatggacgtactttgtctgactacaatattctaaaggactc
taaacttcatcctctgttgagacttcgttaa

Pparg regulatory element reporter (699-EGFP):

TATTCTGTCAACTATTCCTTTTTATAGAATTTGGATAGCAGTAACATTTTGGACCTTTTATAGACTTGTTGAATAAATCACCTTAGAAATGTATTTTGTT
ACCTGTGTGGGTAACAAAATCTAAAATAAGAATGTGTATATGTTTGAGTACAAGAATATTCTTCAGATGTGTGATTAGGAGTTTCAACCAAAGATAA
ATACTTAAGAAAAACTTTGGCCAAATACGTTTATCTGGTGTTTCATAACTTAGAGATTAAGGTTTTCTATTTTAAAAGCCACTGGTGTGTATTTTACTG
CAATTTTAAAAAGCAATCAATATTGAACAATCTCTGCTCTGGTAATTCCAACTACTGTACAGTTCACGCCCCTCACAGAACAGTGAATGTGTGGGTC
ACTGGCGAGACAATGTAGCAACGTTTTCCTTGTAATGTACCAAGTCTTGCCAAAGCAGCAGACAGCATTATGACACACCATTTTGTCACAACTGGCT
CTCAGTCAGGACAGTGCCAGCCAATTCAGGCCTGATTCTTTCTGTGTTTATTCCCACCTCTCCCAAATATTTGAAAACTGGTGTCTTGACTTATTAGC
ATATTCATAAGCTCGATGACCATAAGCCTTTTTCCTTTAACCAACCAATCTTTTGCAAGACATAGACAAAACACCAGTGTGAATTACAGCAAATCTCT
GTTTTATGCTGTTatggtgagcaagggcgaggagctgttcaccggggtggtgcccatcctggtcgagctggacggcgacgtaaacggccacaagttcagcgtgtccggcgagggcgag
ggcgatgccacctacggcaagctgaccctgaagttcatctgcaccaccggcaagctgcccgtgccctggcccaccctcgtgaccaccctgacctacggcgtgcagtgcttcagccgctaccccgac
cacatgaagcagcacgacttcttcaagtccgccatgcccgaaggctacgtccaggagcgcaccatcttcttcaaggacgacggcaactacaagacccgcgccgaggtgaagttcgagggcgaca
ccctggtgaaccgcatcgagctgaagggcatcgacttcaaggaggacggcaacatcctggggcacaagctggagtacaactacaacagccacaacgtctatatcatggccgacaagcagaaga
acggcatcaaggtgaacttcaagatccgccacaacatcgaggacggcagcgtgcagctcgccgaccactaccagcagaacacccccatcggcgacggccccgtgctgctgcccgacaaccact
acctgagcacccagtccgccctgagcaaagaccccaacgagaagcgcgatcacatggtcctgctggagttcgtgaccgccgccgggatcactctcggcatggacgagctgtacaagtaa

Aronesty, E. (2013). Comparison of Sequencing Utility Programs. The Open Bioinformatics Journal 7, 1-8.

Clement, K., Rees, H., Canver, M.C., Gehrke, J.M., Farouni, R., Hsu, J.Y., Cole, M.A., Liu, D.R., Joung, J.K., Bauer, D.E., and Pinello, L. (2019). CRISPResso2 provides accurate and rapid genome editing sequence analysis. Nature Biotechnology 37, 224-226.

Concordet, J.P., and Haeussler, M. (2018). CRISPOR: intuitive guide selection for CRISPR/Cas9 genome editing experiments and screens. Nucleic Acids Res 46, W242-w245.

Fu, T., Sun, W., Xue, J., Zhou, Z., Wang, W., Guo, Q., Chen, X., Zhou, D., Xu, Z., Liu, L., *et al.* (2023). Proteolytic rewiring of mitochondria by LONP1 directs cell identity switching of adipocytes. Nature Cell Biology 25, 848-864.

Hsiau, T., Conant, D., Rossi, N., Maures, T., Waite, K., Yang, J., Joshi, S., Kelso, R., Holden, K., Enzmann, B.L., and Stoner, R. (2019). Inference of CRISPR Edits from Sanger Trace Data. bioRxiv, 251082.

Li, X., Zhang, G., Huang, S., Liu, Y., Tang, J., Zhong, M., Wang, X., Sun, W., Yao, Y., Ji, Q., *et al.* (2023). Development of a versatile nuclease prime editor with upgraded precision. Nat Commun 14, 305.

Magoč, T., and Salzberg, S.L. (2011). FLASH: fast length adjustment of short reads to improve genome assemblies. Bioinformatics 27, 2957-2963.

Qin, W., Kutny, P.M., Maser, R.S., Dion, S.L., Lamont, J.D., Zhang, Y., Perry, G.A., and Wang, H. (2016). Generating Mouse Models Using CRISPR-Cas9-Mediated Genome Editing. Curr Protoc Mouse Biol 6, 39-66.

Zhang, G., Liu, Y., Huang, S., Qu, S., Cheng, D., Yao, Y., Ji, Q., Wang, X., Huang, X., and Liu, J. (2022). Enhancement of prime editing via xrRNA motif-joined pegRNA. Nature Communications 13, 1856.
